# Osmosensing and scaffolding functions of the oligomeric four-transmembrane domain osmosensor Sho1

**DOI:** 10.1038/ncomms7975

**Published:** 2015-04-21

**Authors:** Kazuo Tatebayashi, Katsuyoshi Yamamoto, Miho Nagoya, Tomomi Takayama, Akiko Nishimura, Megumi Sakurai, Takashi Momma, Haruo Saito

**Affiliations:** 1 Division of Molecular Cell Signaling, Institute of Medical Science, The University of Tokyo, 4-6-1 Shirokanedai, Minato-ku, Tokyo 108-8639, Japan; 2 Department of Biological Sciences, Graduate School of Science, The University of Tokyo, Tokyo 113-0033, Japan

## Abstract

The yeast high osmolarity glycerol (HOG) pathway activates the Hog1 MAP kinase, which coordinates adaptation to high osmolarity conditions. Here we demonstrate that the four-transmembrane (TM) domain protein Sho1 is an osmosensor in the HKR1 sub-branch of the HOG pathway. Crosslinking studies indicate that Sho1 forms planar oligomers of the dimers-of-trimers architecture by dimerizing at the TM1/TM4 interface and trimerizing at the TM2/TM3 interface. High external osmolarity induces structural changes in the Sho1 TM domains and Sho1 binding to the cytoplasmic adaptor protein Ste50, which leads to Hog1 activation. Besides its osmosensing function, the Sho1 oligomer serves as a scaffold. By binding to the TM proteins Opy2 and Hkr1 at the TM1/TM4 and TM2/TM3 interface, respectively, Sho1 forms a multi-component signalling complex that is essential for Hog1 activation. Our results illuminate how the four TM domains of Sho1 dictate the oligomer structure as well as its osmosensing and scaffolding functions.

## Abstract (web summary)

The yeast high osmolarity glycerol pathway activates the Hog1 MAP kinase via two branches, SLN1 and SHO, but the identity of the osmosensor has only been shown for the SLN1 branch. Here the authors demonstrate that the four-TM domain protein Sho1 functions as both an osmosensor and adaptor protein necessary for Hog1 activation.

## Graphical abstract

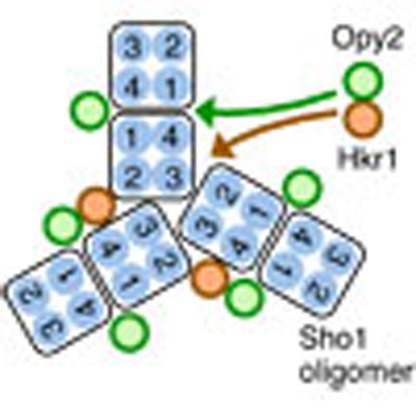

Extreme osmotic environments are major threats to living organisms^1^,^2^. To cope with external high osmolarity, the budding yeast *Saccharomyces cerevisiae* activates the Hog1 MAP kinase (MAPK) through the high osmolarity glycerol (HOG) signalling pathway^3^,^4^. Yeast achieves long-term adaptation to hyperosmotic conditions by accumulating the compatible osmolyte glycerol in the cytoplasm. To do so, activated Hog1 is transported from the cytoplasm to the nucleus^5^, where it induces the expression of the genes that encode the enzymes necessary for glycerol synthesis (Gpd1, Gpp1/2 and so on), and the gene that encodes glycerol/proton symporter Stl1 (refs 6, 7). In the cytoplasm, activated Hog1 closes the glycerol leak channel Fps1 (ref. 8). Thus, Hog1 enhances the production, import and retention of glycerol. Activated Hog1 also regulates the cell cycle progression for optimum adaptation^9^.

The HOG pathway comprises the upstream SLN1 and SHO1 branches, both of which activate the Hog1 MAPK (Fig. 1). The SHO1 branch employs two related, but distinct, signalling mechanisms^10^, which we hereafter call the HKR1 and MSB2 sub-branches. For activation of the HOG pathway, an osmosensor must detect the extracellular osmotic change and subsequently transduce a signal to the cytoplasm. The Sln1 sensor histidine kinase has been firmly established as the osmosensor for the SLN1 branch^11^. However, there has been a controversy regarding the identity of the osmosensor for the SHO1 branch. Three TM proteins, Hkr1, Msb2 and Sho1, have each been posited as putative osmosensors, mainly based on their mutant phenotypes^12^,^13^, but no definitive evidence exists.

Hkr1 and Msb2 share a common function, as it is necessary to disrupt both the *HKR1* and *MSB2* genes to completely inactivate the SHO1 branch^13^. Although both Hkr1 and Msb2 are single-path TM proteins whose extracellular domains contain a highly O-glycosylated Ser/Thr-rich (STR) domain and a conserved Hkr1–Msb2 homology (HMH) domain, their cytoplasmic domains differ. Deletion of the STR domain from either Hkr1 or Msb2 constitutively activates the protein, whereas deletion of the HMH domain inactivates the protein, suggesting that both Hkr1 and Msb2 are involved in signalling.

Sho1 is composed of four TM domains and a cytoplasmic SH3 domain that binds to the MAPK kinase (MAPKK) Pbs2 (ref. 12). Mutations have been identified in the Sho1 TM domains that up- or downregulate osmostress signalling, implying that the Sho1 TM domains actively signal^13^,^14^. However, the finding that deletion of the four TM domains of Sho1 did not completely abolish signalling through the SHO1 branch seemed to contradict the idea that Sho1 might be an osmosensor, since TM signalling would be considered essential for a proposed osmosensor^15^. Follow-up analyses, however, indicated that this Sho1-TM-independent Hog1 activation occurs only through the MSB2 sub-branch^13^. Therefore, the possibility remains that Sho1 serves as an osmosensor for the HKR1 sub-branch. This article investigated the role of Sho1 as an osmosensor in the HKR1 sub-branch.

In response to hyperosmolarity, the HKR1 sub-branch activates Hog1 through the Ste20–Ste11–Pbs2–Hog1 kinase cascade^4^. The PAK-like kinase Ste20 is recruited to the membrane by the small G protein Cdc42 (ref. 15) as well as by Hkr1 (probably through a hypothesized adaptor protein)^10^. Similarly, the MAPKK kinase (MAPKKK) Ste11 is recruited to the membrane by the Opy2–Ste50 complex^16^ (Fig. 2a). Ste50 is a cytoplasmic adaptor protein that binds both to Ste11 and to the single-path membrane anchor protein Opy2 (refs 17, 18). Finally, Pbs2 is also recruited to the membrane by Sho1 (ref. 12). Thus, both the Ste20→Ste11 reaction and the Ste11→Pbs2 reaction take place on the membrane. One or both of these activation reactions are likely regulated by osmostress; however, no such mechanisms are known.

In this article, we demonstrate that Sho1 is an osmosensor in the HKR1 sub-branch of the HOG pathway. Sho1 forms planar oligomers of the dimers-of-trimers architecture by dimerizing at the TM1/TM4 interface and trimerizing at the TM2/TM3 interface. High external osmolarity induces structural changes in the Sho1 TM domains and Sho1 binding to the cytoplasmic adaptor protein Ste50, which leads to Hog1 activation. Furthermore, by binding to the TM proteins Opy2 and Hkr1 at the TM1/TM4 and TM2/TM3 interface, respectively, Sho1 forms a multi-component signalling complex that is essential for Hog1 activation. Thus, the four TM domains of Sho1 dictate the oligomer structure as well as its osmosensing and scaffolding functions.

## Results

### Osmostress induces the Ste50–Sho1 association

**Figure 2:**
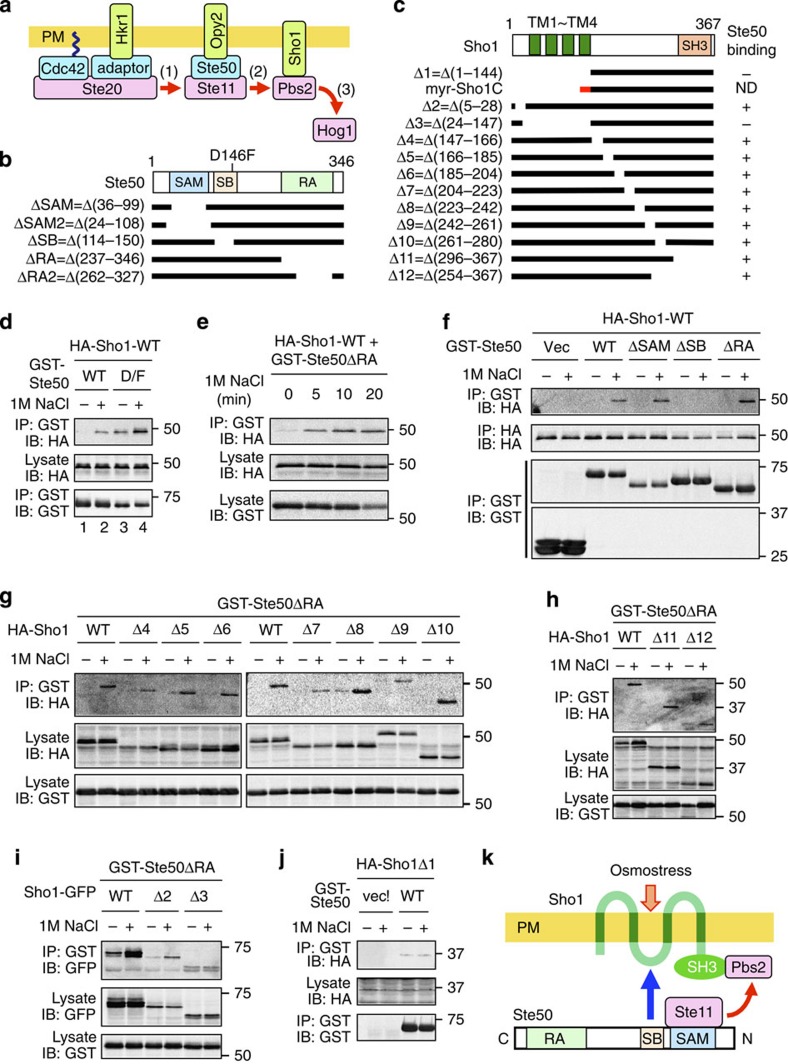
Osmostress induces Ste50–Sho1 association. (**a**) A simplified schematic model of the HKR1 sub-branch of the HOG pathway. Red arrows indicate the flow of signal by sequential phosphorylation. The yellow horizontal bar represents the plasma membrane (PM). (**b**) Schematic model of Ste50. Horizontal black bars represent the Ste50 deletion constructs used in this work. RA, Ras association domain; SAM, sterile alpha motif; SB, Sho1-binding domain. (**c**) Schematic model of Sho1. Horizontal black bars represent the Sho1 deletion constructs. The short red bar represents the myristoylation (myr) site. ND, not determined. (**d**–**j**) Ste50–Sho1-binding assays. The yeast strain KY594-1 was co-transfected with the expression plasmids for indicated derivatives of HA-Sho1 (**d**–**h**,**j**) or Sho1-GFP (**i**) under the *GAL1* promoter, and for GST-Ste50 under the *TEF2* promoter. Cells were grown in CARaf, and expression of tagged Sho1 was induced by 2% galactose for 2 h. NaCl (1 M, final conc.) was added (+) for 5 min or for the times indicated in (**e**), or was not added (−), and cell lysates were prepared using Buffer A containing 0.2% Triton-X-100. GST-Ste50 was precipitated from cell lysates with glutathione Sepharose, and co-precipitated HA-Sho1 or Sho1-GFP was probed with an anti-HA (**d**–**h**,**j**) or an anti-GFP (**i**) antibody, respectively. IB, immunoblotting; IP, immunoprecipitation. (**k**) A model of the osmostress-induced Ste50–Sho1 interaction. The blue arrow indicates inducible physical association, whereas the red arrows indicate the flow of signal.

To see if Sho1 potentially functions as an osmosensor, we first examined if osmostress might regulate Sho1 interaction with any signalling molecule of the SHO1 pathway. We thought the adaptor protein Ste50 especially a likely candidate, as we previously showed that a hyperactive Ste50-D146F, but not the wild-type Ste50, binds to Sho1 (ref. 19). Hyperactive mutants often mimic stimulus-induced activation states of their wild-type counterparts. We therefore tested if osmostress could induce wild-type Ste50–Sho1 interaction using co-immunoprecipitation assays (see Fig. 2b,c for schematic structures of Ste50 and Sho1 constructs). Although GST-Ste50-WT did not bind to HA-Sho1 in the absence of osmostress, it substantially bound to HA-Sho1 when the cells were exposed to osmostress, such as 1.0 M NaCl (Fig. 2d, lanes 1–2). The binding between GST-Ste50-D146F and HA-Sho1, which was detectable even in the absence of osmostress, was further strengthened by osmostress (Fig. 2d, lanes 3–4). As we will show later in this paper, Sho1 also interacts with Opy2. To prevent an indirect association of Sho1 and Ste50 mediated by Opy2, we used the Ste50ΔRA mutant, which cannot bind to Opy2, for Ste50–Sho1-binding assays in the remainder of this work. Osmostress-induced binding between GST-Ste50ΔRA and HA-Sho1 occurred rapidly, reaching a plateau in 10 min (Fig. 2e). These data therefore indicate that osmostress does induce the interaction of Sho1 with the adaptor protein Ste50. Induced Ste50–Sho1 association may enhance Ste11–Pbs2 interaction, which is an essential step in the Hog1 MAPK cascade (step 2 in Fig. 2a).

### A conserved domain in Ste50 binds to the Sho1 TM domains

We next addressed the Ste50–Sho1-binding sites within these molecules. Ste50 contains three evolutionarily conserved domains—the amino-terminal (N-terminal) SAM domain that binds Ste11, a central conserved region (amino-acid positions 114–150) of unknown function and the carboxyl-terminal (C-terminal) RA domains that bind Opy2 (see Fig. 2b)^16^,^17^,^19^. Using GST-Ste50 constructs that lacked one of these domains, we found that the central conserved region was necessary for the osmostress-induced binding of GST-Ste50 to HA-Sho1 (Fig. 2f). We hereafter refer to the central conserved region of Ste50 as the Sho1-binding (SB) domain.

To identify the site in Sho1 that bound to Ste50, we similarly examined the binding of a set of HA-Sho1 partial deletion mutants to GST-Ste50ΔRA (see Fig. 2c for mutants used). Osmostress-induced Ste50–Sho1 binding was unaffected by deletion of any part of the Sho1 C-terminal (Fig. 2g–h, Sho1Δ4–Δ12) or N-terminal (Fig. 2i, Sho1Δ2) cytoplasmic regions, but was abolished by deletion of the four Sho1 TM domains (Fig. 2i, Sho1Δ3; and Fig. 2j, Δ1). We therefore concluded that the four Sho1 TM domains, but no other parts of Sho1, are necessary for its osmostress-induced Ste50 binding.

### Induced Ste50–Sho1 binding activates Hog1

**Figure 3:**
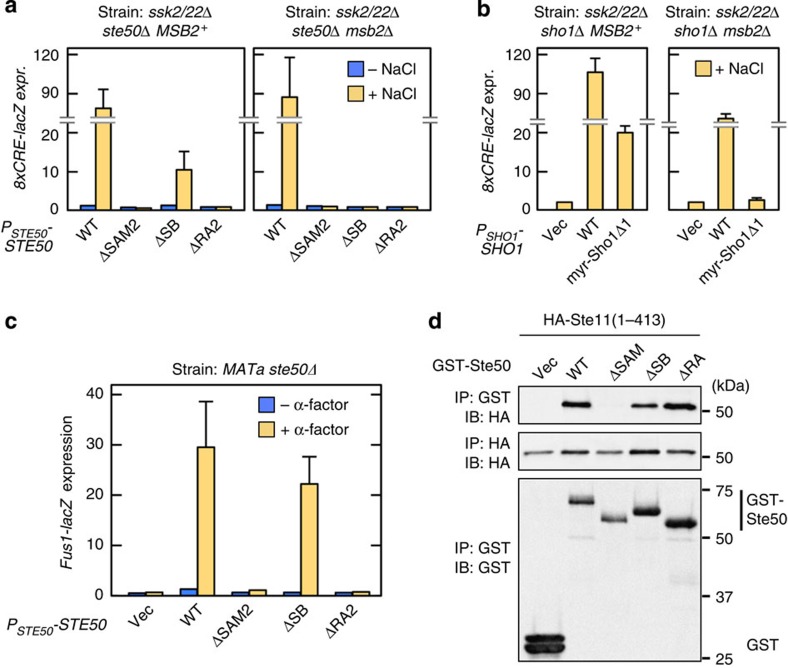
Role of the Sho1-binding (SB) domain of Ste50 in Hog1 activation. (**a**,**b**) Hog1-specific reporter assays of yeast strains of the indicated genotypes; KT048 and KT193 in **a** or QG153 and KT040 in **b**, which were transfected with expression plasmids for the indicated mutants of *STE50* (**a**) or *SHO1* (**b**). Cells were stimulated with (orange) or without (blue) 0.4 M NaCl for 30 min, and expression (expr.) of the *8xCRE-lacZ* reporter gene was determined. β-galactosidase activity is expressed in Miller units. Error bars represent s.d. (*n*⩾3). (**c**) Reporter assay for the pheromone-responsive Fus3/Kss1 MAPK pathway. FP66 was co-transformed with the Fus3/Kss1-specific reporter plasmid pFUS1-lacZ and expression plasmids for the indicated Ste50 mutants. Cells were stimulated with or without 1 μM α-factor for 2 h, and expression of the *FUS1-lacZ* reporter gene was determined. Error bars represent s.d. (*n*⩾3). (**d**) *In vivo* Ste50–Ste11-binding assay. The yeast strain KY594-1 was co-transfected with expression plasmids for indicated derivatives of GST-Ste50 and HA-Ste11ΔC (=1–413) under the *GAL1* promoter. Cells were grown in CARaf, and expression of GST-Ste50 and HA-Ste11ΔC was induced by 2% galactose for 2 h. Cell lysates were prepared using Buffer A containing 0.2% Triton-X-100. GST-Ste50 was precipitated from cell lysates with glutathione Sepharose, and co-precipitated HA-Ste11ΔC was probed with an anti-HA antibody.

We next determined if Ste50–Sho1 binding might be essential for Hog1 activation. Ste50ΔSB, which lacked the Sho1-binding site, could support substantial Hog1 activation in *ssk2/22*Δ *MSB2*^+^ cells, but not in *ssk2/22*Δ *msb2*Δ cells (Fig. 3a). Similarly, a Sho1 mutant that lacked the Ste50-binding site, namely myr–Sho1Δ1, supported Hog1 activation in *ssk2/22*Δ *MSB2*^+^ cells but not in *ssk2/22*Δ *msb2*Δ cells (Fig. 3b). In this experiment, a myristoylation (myr) signal was attached at the N terminus of Sho1Δ1 so that it could recruit the bound Pbs2 to the membrane^15^. In contrast to these data, the *ste50*ΔSB mutation did not inhibit the pheromone-responsive Fus3/Kss1 MAPK pathway that also involves Ste50 signalling^20^ (Fig. 3c). Consistent with these results, GST-Ste50ΔSB could bind to Ste11, which is required both for the HKR1 sub-branch and for the mating pathway (Fig. 3d). On the basis of these results, we conclude that osmostress-induced Ste50–Sho1 binding is essential specifically for Hog1 activation via the HKR1 sub-branch (but neither for the MSB2 sub-branch nor for the mating pathway).

### Membrane permeabilization induces Ste50–Sho1 association

**Figure 4:**
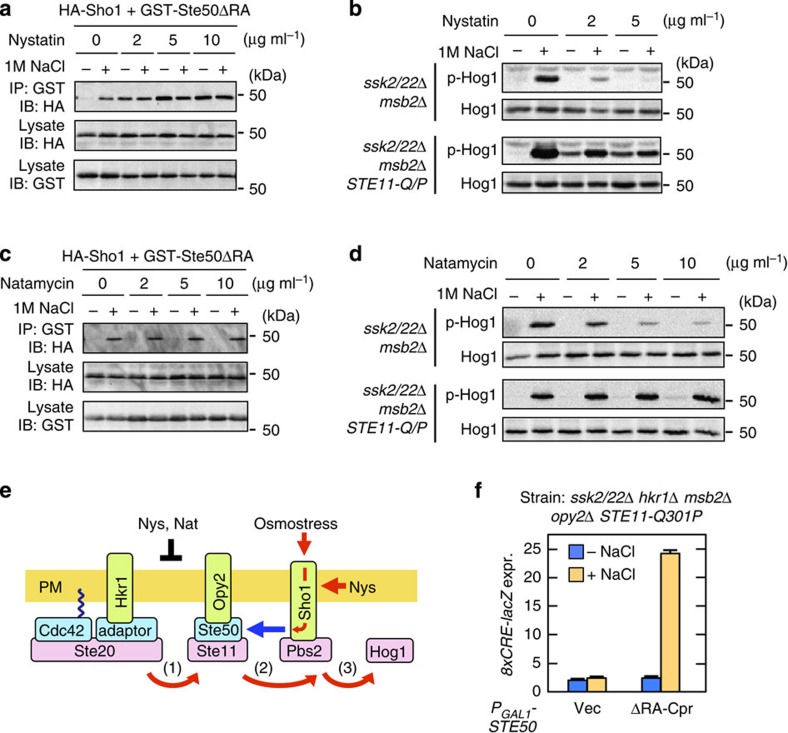
Membrane permeabilization activates the Sho1 osmosensor. (**a**) Ste50–Sho1-binding assays. The procedure was the same as in Fig. 2d, except that the cells were treated with the indicated concentrations of nystatin for 5 min immediately before 1 M NaCl (final concentration) was added. (**b**) Hog1 phosphorylation assays. The yeast strains KT034 and KT033, of the indicated genotypes, were treated with the indicated concentration of nystatin for 5 min, and were then stimulated with (+) or without (−) 1 M NaCl for a further 5 min. Hog1 phosphorylation was determined by immunoblotting using an anti-phospho-p38 antibody. (**c**,**d**) The same as in **a** and **b**, respectively, except that natamycin was used in place of nystatin. (**e**) Model of the induced Ste50–Sho1 interaction. Red arrows, positive flow of the signal; black bar, inhibition of signalling; blue arrow, inducible protein binding; Nat, Natamycin; Nys, Nystatin. (**f**) Hog1-specific reporter assays of KY594-1 cells transformed with an expression plasmid for the Ste50ΔRA-Cpr construct. Ste50ΔRA-Cpr was expressed from the *GAL1* promoter for 2 h. Expression of the *8xCRE-lacZ* reporter gene was determined as in Fig. 3a. Error bars indicate s.d. (*n*=3).

Osmostress exerts diverse physical and chemical effects on the cell. To determine which of these effects of osmostress induced Sho1 to bind Ste50, we surveyed a number of pharmacological and toxicological agents that might mimic the effects of osmostress. Of these agents, the antifungal drug nystatin was of special interest, because it had previously been shown to inhibit the SHO1 branch^21^, and was therefore expected to inhibit osmostress induction of the Ste50–Sho1 association. However, in contrast to our expectations, nystatin induced strong Ste50–Sho1 association even in the absence of osmostress (Fig. 4a). To better understand this effect of nystatin, we re-examined its effects on steps of Hog1 activation. In an *ssk2/22*Δ *msb2*Δ mutant strain, in which only the HKR1 sub-branch is functional, 5 μg ml^−1^ nystatin completely inhibited the osmostress-induced Hog1 activation (Fig. 4b, upper rows). However, in a corresponding strain in which the hyperactive *STE11*-Q301P was expressed (from the native *STE11* promoter), nystatin activated Hog1 even in the absence of osmostress (Fig. 4b, lower rows). To interpret these results, we considered that two steps in the Hog1 MAPK cascade, Ste20→Ste11 (step 1 in Fig. 2a) and Ste11→Pbs2 (step 2), are likely be regulated by osmostress to effect Hog1 activation^19^. The Ste11-Q301P mutant protein mimics the activated state of Ste11, and thus renders step 1 unnecessary. Thus, a plausible interpretation of these results is that nystatin stimulates step 2, but inhibits step 1.

We next determined how nystatin induced Sho1–Ste50 interaction. Nystatin exerts its antifungal effects by two distinct mechanisms—sequestration of ergosterol and membrane permeabilization^22^. To distinguish which of these effects might activate step 2, we studied the structurally related natamycin, which binds to ergosterol but does not permeabilize the membrane^22^. Unlike nystatin, natamycin did not induce the binding of GST-Ste50ΔRA to HA-Sho1 (Fig. 4c), and did not activate Hog1 in *STE11*-Q301P cells (Fig. 4d, lower rows). However, similar to nystatin, natamycin did inhibit Hog1 activation in *STE11*-WT cells (Fig. 4d, upper rows). Thus, we concluded that the inhibition of Hog1 activation by both drugs is due to their ergosterol binding and that induction of the Ste50–Sho1 interaction by nystatin is caused by membrane permeabilization (Fig. 4e).

In bacteria, the N- and C-terminal cytoplasmic domains of several osmosensors, such as the betaine transporter BetP, respond to increased concentration of K^+^ in the cytoplasm^23^. To examine if the Sho1 osmosensor also responds to changes in the cytoplasmic concentration of specific ions, such as H^+^ or K^+^, we treated yeast cells (expressing the hyperactive STE11-Q301P kinase) with the proton ionophore carbonylcyanide-3-chlorophenylhydrazone or with the potassium ionophore valinomycin^24^. However, they neither activated Hog1 in the absence of osmostress, nor inhibited Hog1 activation in the presence of osmostress (Supplementary Fig. 1a). In contrast, enzymatic digestion of the cell wall by zymolyase potently activated Hog1 if the cells carried the Ste11-Q301P mutation (Supplementary Fig. 1b). Because these treatments (membrane permeabilization, digestion of the cell wall and extracellular high osmolarity) reduce turgor^21^, Sho1 might respond to reduced turgor. It is also possible, however, that Sho1 responds to changes in other properties of the plasma membrane, such as lateral pressure or viscosity.

### Sho1 is a functional osmosensor

To provide formal proof that Sho1 is by itself an osmosensor, rather than a cofactor of another TM osmosensor, we investigated if Sho1 can activate Hog1 in response to osmostress in the absence of Hkr1, Msb2 and Opy2. Normally, no trace of Hog1 activation can be detected in the absence of Hkr1, Msb2 and Opy2 (in an *ssk2/22*Δ background). However, to test our Sho1 osmosensor proposal, we created cells in which the need for these membrane proteins for osmostress-induced Hog1 activation was bypassed. For this purpose, we first expressed hyperactive Ste11-Q301P (from its own promoter) to at least partially circumvent the need for Hkr1 and Msb2, whose major function is to help Ste20 activate Ste11 (ref. 10). Second, we artificially localized Ste50 to the membrane by attachment of a C-terminal prenylation (Cpr) signal, which circumvents, again at least partially, the need for Opy2 (ref. 13). By combining these conditions, it was indeed possible to activate Hog1 by osmostress in the absence of Hkr1, Msb2 and Opy2 (Fig. 4f). Thus, we concluded that Sho1 is an osmosensor proper.

### Sho1 oligomerizes by the dimers-of-trimers architecture

**Figure 5:**
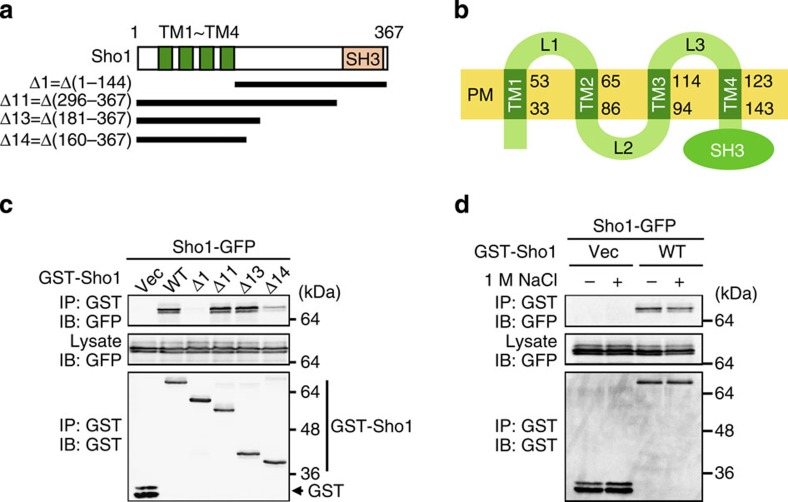
Sho1 homo-oligomerization requires the TM domains. (**a**) The Sho1 deletion constructs used in **c**. TM, transmembrane domains. (**b**) Schematic model of Sho1. Numbers are amino-acid positions. L, Loop region. (**c**,**d**) Sho1–Sho1-binding assays. p424GAL1-GST-Sho1 (or its deletion derivative as indicated) and p416GAL1-Sho1-GFP were co-expressed in KT079 (*sho1*Δ) cells. GST-Sho1 was affinity purified using glutathione beads, and co-precipitated Sho1-GFP was detected by immunoblotting. (**d**) 1 M NaCl was added as indicated for 5 min before preparation of cell lysates.

To understand how Sho1 transmits a signal across the membrane, we first studied the structure of Sho1. Since Sho1 has been reported to homo-oligomerize^25^, we examined which region of Sho1 is required for oligomerization, by analysis of the co-precipitation of full-length Sho1-green fluorescent protein (GFP) with various partial deletion constructs of GST-Sho1 (Fig. 5a,b). Sho1Δ1, which lacks all four TM domains, could not oligomerize at all, whereas Sho1Δ14, which contains only the four TM domains and their immediate flanking regions, could oligomerize, albeit weakly (Fig. 5c). Thus, the four TM domains are responsible for Sho1 oligomerization. The extent of Sho1 oligomerization was not altered by external high osmolarity (Fig. 5d).

**Figure 6:**
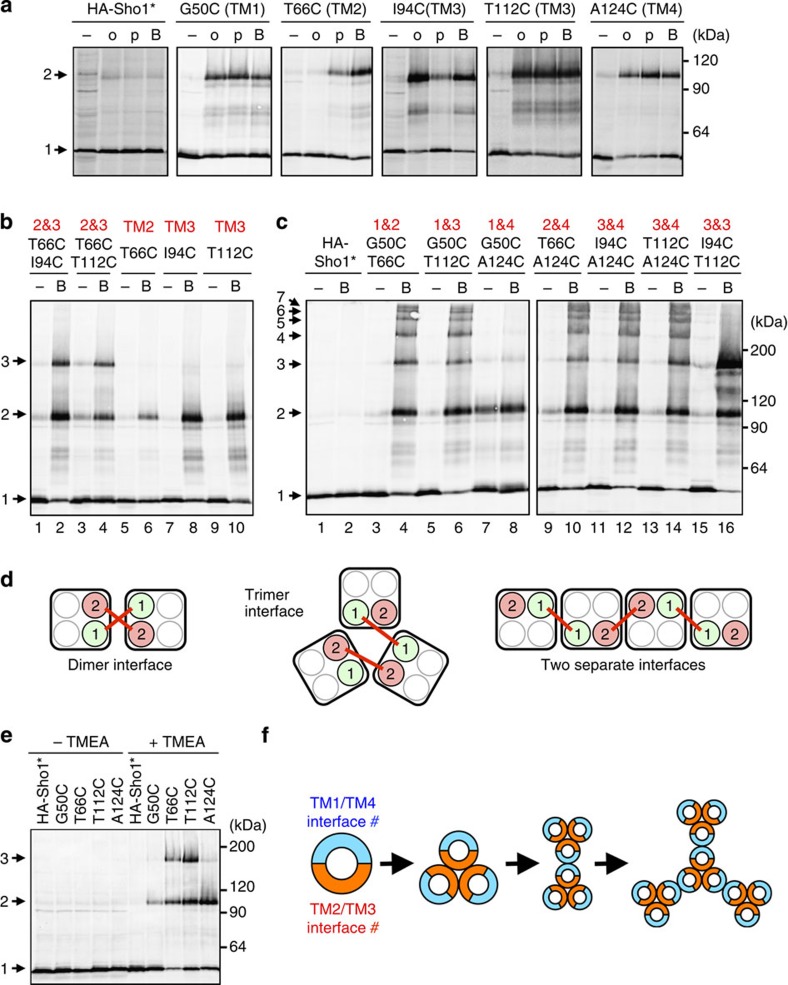
Chemical crosslinking analyses of Sho1 oligomers. (**a**–**c**) Chemical crosslinking of Sho1 single- or double-Cys substitution mutants. The indicated mutant derivatives of the Cys-less HA-Sho1* were individually expressed in KT079 cells, and isolated membrane fractions were treated with the indicated crosslinkers *in vitro*. Samples were subjected to SDS–PAGE with 2-ME and HA-Sho1* was detected by immunoblotting. Numbers on the right indicate the extent of Sho1 polymerization (1, monomer; 2, dimer and so on). Numbers in red indicate the TM domain location of the Cys mutations. o, *o*-PDM; p, *p*-PDM; B, BMH. (**d**) Schematic models for interpretation of the results of the double-Cys crosslinking experiments (see text). (**e**) Chemical crosslinking of Sho1 single-Cys substitution mutants with the homo-trifunctional crosslinker TMEA. The procedure was the same as in **a**. (**f**) Schematic model of Sho1 oligomerization. Sho1 is represented by a circle, with the dimeric TM1/TM4 interface indicated in blue and the trimeric TM2/TM3 interface in orange. A potential oligomerization route is shown, but other intermediates and final structures are also possible.

To determine which of the four TM domains participated in Sho1–Sho1 binding, we used a site-directed cysteine (Cys) chemical crosslinking strategy^26^. As Sho1-WT contains two native Cys residues, we first generated a Cys-less (C78S C85S) mutant (termed Sho1*), which was functional (Supplementary Fig. 2a). We then constructed a collection of single-Cys substitution mutants of HA-tagged Sho1* (HA-Sho1*), by individually mutating to Cys at almost all positions in the four TM domains (see Fig. 5b). A detergent-free membrane fraction was then prepared from *sho1*Δ cells in which single-Cys mutants were individually expressed. The isolated membrane was treated with bifunctional thiol crosslinkers of different lengths; *N*,*N*'-*o*-phenylenedimaleimide (*o*-PDM; 6 Å), *N*,*N'*-*p*-phenylenedimaleimide (*p*-PDM; 10 Å) or 1,6-*bis*-maleimidohexane (BMH; 6–16 Å)^26^. There were several residues in each TM domain that could be homo-crosslinked (Fig. 6a, Supplementary Fig. 2b). Since Sho1 crosslinking occurred efficiently at 0 °C (Supplementary Fig. 2c), which is well below the lipid phase transition point, the observed crosslinking was between stably associated Sho1 molecules, rather than between randomly collided molecules^27^. We therefore concluded that each TM domain of one Sho1 molecule is physically close to the same TM domain in an adjacent Sho1.

To determine how one Sho1 abutted against another Sho1, we constructed double-Cys mutants. Sho1 mutants that have one Cys in TM2 and another in TM3 (T66C I94C and T66C T112C) formed trimers (and dimers) on crosslinking (Fig. 6b, lanes 2 and 4). A Sho1 mutant that has two Cys in TM3 (I94C T112C) also formed trimers (Fig. 6c, lane 16). In contrast, Sho1 mutants that have one Cys in TM1 and another in TM4 (G50C A124C) formed only dimers (Fig. 6c, lane 8). Sho1 mutants with other combinations of two Cys generated a ladder of higher-order oligomers (Fig. 6c). The models in Fig. 6d show how the arrangements of TM domains dictate the outcomes of double-Cys crosslinking. If the two TM domains that contain Cys substitutions are at a common dimer interface, then their crosslinking will generate covalent dimers (Fig. 6d, left). This was the case when TM1 and TM4 were crosslinked. If, on the other hand, the two Cys substitutions are at a trimer interface, crosslinking will generate covalent trimers (Fig. 6d, middle). This was the case when TM2 and TM3 were crosslinked. Finally, if the two Cys substitutions belong to different interfaces, crosslinking will generate higher-order oligomers (Fig. 6d, right). This was the case for all combinations other than TM1/TM4 and TM2/TM3.

We also probed the structure of Sho1 oligomers using the homo-trifunctional crosslinker tris-(2-maleimidoethyl)amine (TMEA)^28^. Crosslinking of single-Cys substitution mutants T66C (TM2) or T112C (TM3) by TMEA formed trimers, whereas crosslinking of G50C (TM1) or A124C (TM4) formed only dimers (Fig. 6e). These results confirmed that Sho1 trimerizes at the TM2/TM3 interface, whereas it dimerizes at the TM1/TM4 interface. In other words, Sho1 oligomerizes by repetitive ‘dimers-of-trimers' architecture (Fig. 6f).

### The arrangements of TM domains within the two interfaces

**Figure 7:**
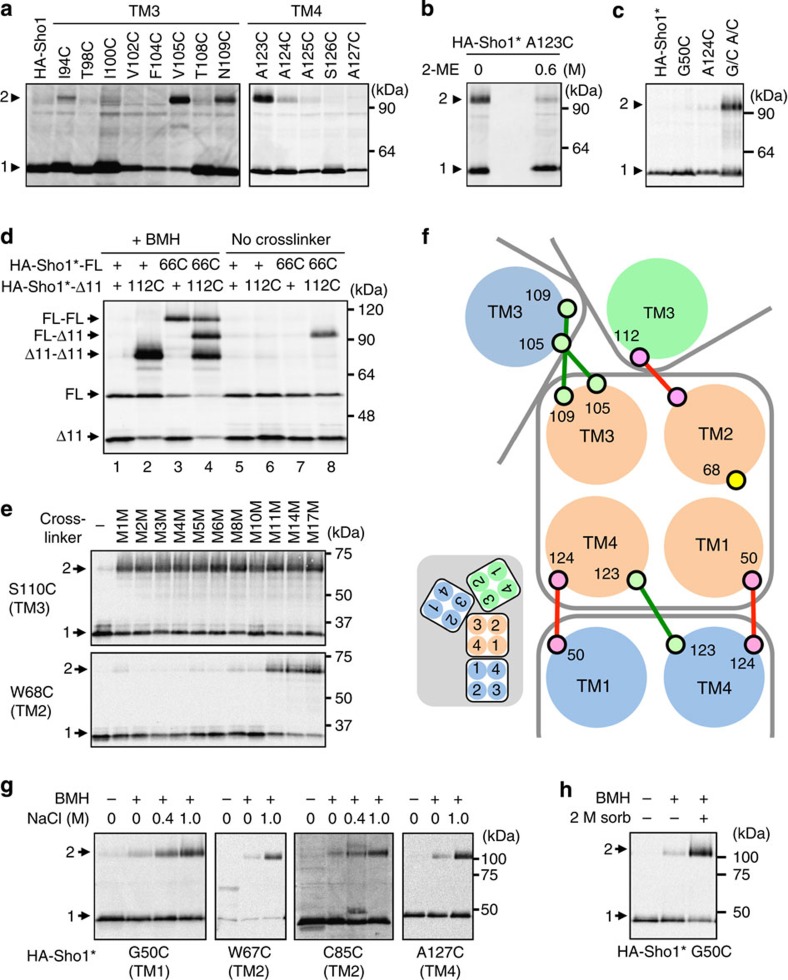
Arrangements of the four TM domains in the Sho1 oligomers and osmostress-induced structural changes in Sho1. (**a**) Spontaneous disulfide bond formation by Sho1 single-Cys substitution mutants. Membrane fractions were subjected to SDS–PAGE (without 2-ME) and HA-Sho1 and its mutant derivatives were detected by immunoblotting. (**b**) Inhibition of disulfide bond formation by the reductant 2-ME. Sample lanes are intentionally separated by two empty lanes. (**c**) Spontaneous disulfide bond formation by a Sho1 double-Cys substitution mutant. G/C, G50C; A/C, A124C. (**d**) Spontaneous disulfide bond formation between two Sho1 single-Cys substitution mutants. Membrane fractions were treated with (+) or without (−) BMH and were subjected to SDS–PAGE (without 2-ME). 66C, T66C; 112C, T112C; Sho1Δ11 is Sho1Δ(296–367). (**e**) Chemical crosslinking by molecular ruler. Single-Cys substitution mutants of HA-Sho1*Δ11 were chemically crosslinked using a set of crosslinkers with different spacer lengths (M1M–M17M). The procedure was the same as in Fig. 6a, except that SDS–PAGE was done under non-reducing conditions. (**f**) Model of helix packing at the TM1/4 and TM2/3 interfaces (top view from the outside). Large circles represent TM helices and small circles are amino-acid residues. Red and green lines represent spontaneously formed disulfide bonds. Inset is a smaller scale view of the dimeric and trimeric complexes. (**g**) Osmostress-induced changes in chemical crosslinking of Sho1. KT079 was transformed with expression plasmids for the indicated mutants of HA-Sho1*. HA-Sho1* was induced by 2% galactose for 2 h, and was crosslinked in intact cells with BMH, in the presence or absence of NaCl, as indicated. (**h**) Effects of sorbitol on Sho1 crosslinking. HA-Sho1* G50C was expressed in KT079 and crosslinked as in **a** except in the presence (+) and absence (−) of 2 M sorbitol.

When two Cys residues are sufficiently close to each other, they can form a disulfide bond^29^. Among the single-Cys mutants, I53C, S54C, V105C, N109C and A123C formed spontaneous homodimers under non-reducing conditions (Fig. 7a and Supplementary Fig. 3a). These dimers were disulfide bonded, because dimerization was inhibited by the reductant 2-mercaptoethanol (2-ME) and enhanced by the oxidant copper/phenanthroline (Fig. 7b and Supplementary Fig. 3b).

Disulfide bonding could also occur between the two different Cys residues. In fact, it can be seen in Fig. 6c (lane 7) that the G50C A124C double mutant formed spontaneous dimers without chemical crosslinking. Because neither G50C nor A124C formed disulfide bonds, G50C and A124C must have formed a hetero-disulfide bond (Fig. 7c). Screening of selected double-Cys mutants revealed that the T66C T112C double mutant spontaneously formed disulfide-bonded trimers (Supplementary Fig. 3c). Neither the T66C nor the T112C single-Cys mutant homo-dimerized. To prove more directly that disulfide bonds were formed only between heterologous combinations, we generated full-length Sho1-FL-T66C and truncated Sho1-Δ11-T112C, which are distinguishable by their sizes. In the absence of chemical crosslinker, dimers are formed only between T66C and T112C (Fig. 7d, lanes 6–8). In contrast, chemical crosslinking was also possible between two T66C or between two T112C (Fig. 7d, lanes 2–4). Thus, T66 (TM2) and T112 (TM3) are very close to each other.

We measured the distances between two TM2 domains or two TM3 domains at the TM2/TM3 trimeric interface using a ‘molecular ruler'. This molecular ruler is composed of a set of bifunctional crosslinkers of different lengths (from the shortest, M1M (3.9 Å) to the longest, M17M (24.7 Å))^30^. Thus, we found that the TM3–TM3 distance is much shorter than the TM2–TM2 distance. For example, S110C in TM3 could be crosslinked by the shortest crosslinker M1M, whereas W68C in TM2 could be efficiently crosslinked only by M11M (16.9 Å) or a longer crosslinker (Fig. 7e and Supplementary Fig. 3d). On the basis of these analyses, a plausible arrangement of the four TM domains was constructed as shown in Fig. 7f.

### Osmostress induces structural changes in the Sho1 TM domains

We next asked how Sho1 transmits the osmotic signal across the plasma membrane. In general, cell surface receptors transmit signals either by ligand-induced dimerization or by ligand-induced conformational changes in their TM domains. As we could find no evidence that the extent of Sho1 oligomerization was altered by external high osmolarity, we examined if osmostress would induce structural changes in the Sho1 TM domains. Structural changes in the TM domains will alter the crosslinking efficiencies of the moved residues, either by changing the distance between two Cys residues, or by changing the accessibility of the crosslinking reagent. We found that the efficiency of crosslinking of HA-Sho1* G50C, W67C, C85C (the native Cys at 85) and A127C mutants was enhanced by osmostress, in an osmostress dose-dependent manner (Fig. 7g). Enhanced crosslinking of these mutants was induced by sorbitol as well as by NaCl (Fig. 7h), indicating that osmostress, not a specific solute, is responsible. The enhancement of crosslinking was not due to increased subunit association, as crosslinking at many other positions was unaffected by osmostress (Supplementary Fig. 4a). This crosslinking was inhibited at low temperature (Supplementary Fig. 4b), suggesting that membrane fluidity was essential for the osmostress-induced structural changes (it should be noted that the crosslinking reaction itself can proceed efficiently at 0 °C; see Supplementary Fig. 2c). Osmostress-induced crosslinking of Sho1 could occur in the absence of the other TM proteins of the SHO1 branch, namely Hkr1, Msb2 and Opy2 (Supplementary Fig. 4c,d), implying that the Sho1 structural changes are autonomous responses of Sho1. How the Sho1 structural changes relate to the induced Ste50–Sho1 binding is unknown.

### The TM1/TM4 interface is important for osmosensing

**Figure 8:**
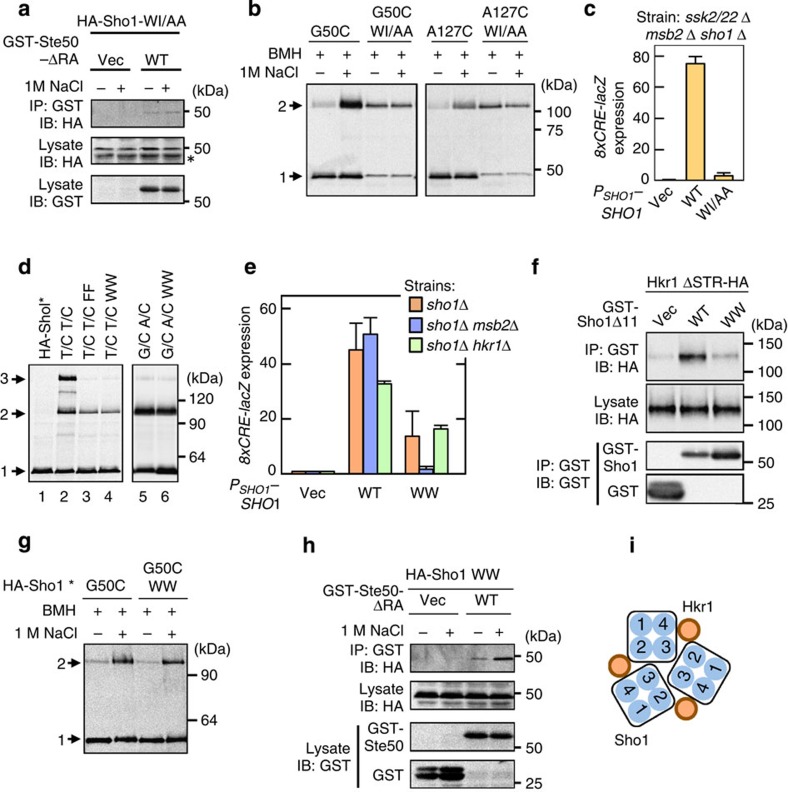
Effects of Sho1 TM domain mutants on Hog1 activation. (**a**) Ste50–Sho1-binding assays were conducted as in Fig. 2d. WI/AA, W139A I140A. (**b**) Sho1 crosslinking assays in the presence or absence of osmostress were conducted as in Fig. 7g. (**c**) Hog1-specific reporter assays of the KT053 strain transformed with the indicated Sho1 expression plasmids. Cells were stimulated with 0.4 M NaCl for 30 min, and expression of the *8xCRE-lacZ* reporter gene was determined. Error bars indicate s.d. (*n*=3). (**d**) Mutants that disrupt the TM2/TM3 interface. The indicated mutants of HA-Sho1* were expressed in KT079 cells, and spontaneous disulfide bond formation was analysed as in Fig. 7a. T/C T/C, T66C T112C; WW, V105W N109W; FF, V105F N109F. (**e**) Hog1-specific reporter assays. KT079, KT053 and KT088 cells were transformed with the indicated expression plasmids for Sho1. The *8xCRE-lacZ* reporter assay was conducted as in **c**. Error bars indicate s.d. (*n*=3). (**f**) Hkr1–Sho1-binding assay. The indicated Hkr1 and Sho1 constructs were co-expressed in KT075. Cell lysates were prepared with Buffer A containing 1% digitonin and binding was assayed as in Fig. 2d. (**g**) Sho1-crosslinking assays were conducted as in Fig. 7g. (**h**) Ste50–Sho1-binding assays were conducted as in Fig. 2d. (**i**) Schematic model of the Sho1–Hkr1 interaction. Blue circles with numbers represent the four TM domains of Sho1.

We next examined the role of the Sho1 oligomeric structure in osmosensing and signalling. For this purpose, we screened for Sho1 mutants that are defective in Ste50 binding, using a collection of Sho1 mutants in which two consecutive amino acids were substituted by other amino acids. We found that HA-Sho1-W139A I140A (WI/AA), which was mutated in the TM4 domain, could not bind GST-Ste50 (Fig. 8a), and did not show enhanced crosslinking efficiency (Fig. 8b), in response to osmostress. Importantly, Sho1-WI/AA could not activate Hog1 via the HKR1 sub-branch (Fig. 8c). The presence of a TM4 mutant that cannot respond to osmostress suggests that the TM1/TM4 interface is important for osmosensing. However, because Sho1-WI/AA could dimerize, it remains unclear whether dimerization is necessary for osmosensing or not.

### The TM2/TM3 trimer interface binds to Hkr1

To examine if the TM2/TM3 interface is required for osmosensing, we created Sho1 mutants that are defective in association via the TM2/TM3 interface. Specifically, we mutated V105 and N109, which are both closely packed at the TM2/TM3 interface (see Fig. 7f), to the bulky Phe or Trp. Both V105F N109F (FF) and V105W N109W (WW) mutations strongly reduced trimerization of Sho1 at the TM2/TM3 interface (as detected by T66C T112C disulfide bonding; Fig. 8d, lanes 3 and 4). The Sho1-WW mutation had no adverse effect on dimerization at the TM1/TM4 interface (as detected by G50C A124C disulfide bonding; Fig. 8d, lane 6). Thus, Sho1-WW specifically disrupts the TM2/TM3 interface without affecting the TM1/TM4 interface.

Sho1-WW could not activate Hog1 in *ssk2/22*Δ *msb2*Δ mutant cells, whereas it could in *ssk2/ssk22*Δ *hkr1*Δ cells, indicating that the HKR1 sub-branch is defective in cells expressing this mutant (Fig. 8e, and Supplementary Fig. 5a,b). Consistent with this interpretation, Sho1-WW could not bind Hkr1, whereas Sho1-WT could bind Hkr1 (ref. 13; Fig. 8f). However, in response to osmostress, the HA-Sho1-WW mutant did undergo structural changes in the TM domains (Fig. 8g) and did associate with GST-Ste50 (Fig. 8h). Thus, Sho1 trimerization at the TM2/TM3 interface is important for the Sho1–Hkr1 interaction (Fig. 8i) and for the signalling function of Sho1, but not for the osmosensing function of Sho1.

### The TM1/TM4 dimer interface binds to Opy2

**Figure 9:**
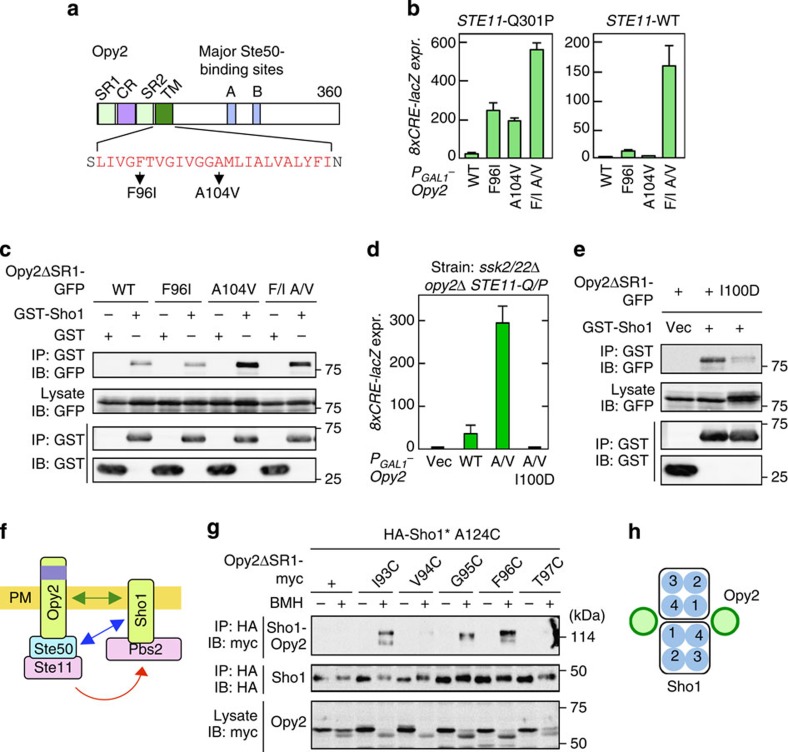
Opy2–Sho1 interaction. (**a**) Schematic structure of Opy2. The amino-acid sequence of the TM domain is shown in red using the one-letter code. (**b**) Hog1-specific reporter assays. KY517 and KY477 were transformed with expression plasmids for the indicated Opy2 mutants. Opy2 mutants were expressed from the *GAL1* promoter for 2 h, and expression of *8xCRE-lacZ* was determined in the absence of osmostress. Error bars indicate s.d. (*n*⩾3). (**c**) Opy2–Sho1-binding assays. TM257 was co-transfected with the indicated derivatives of pRS426GAL1-GST-Sho1 and pRS414GAL1-Opy2-GFP. Expression of the Opy2 and Sho1 constructs were induced by 2% galactose for 2 h, cell lysates were prepared with Buffer A containing 0.3% Brij-35 and binding was assayed as in Fig. 2i. (**d**) Hog1-specific reporter assays of KY517 cells transformed with expression plasmids for the indicated Opy2 mutants. Assays were conducted as in **b**. Error bars indicate s.d. (*n*=3). (**e**) Opy2–Sho1-binding assays were conducted as in **c**. (**f**) Schematic model of the Opy2–Sho1 interaction. Green arrow, physical association; blue arrow, inducible physical association; red arrow, signal flow. (**g**) Chemical crosslinking of Sho1 to Opy2. Expression plasmids (*GAL1* promoter) for the indicated mutant derivatives of Opy2-myc and HA-Sho1*-A124C were co-transfected into KY590-1. After induction by 2% galactose for 2 h, intact cells were treated with 0.4 mM BMH at 30 °C for 5 min. Cell lysates were prepared with buffer A containing 0.2% Triton-X-100. HA-Sho1* was immunoprecipitated, and co-precipitated (covalently bound) Opy2-myc was detected by immunoblotting. (**h**) Schematic model of the Opy2–Sho1 interaction.

We next considered if Sho1 might also interact with the other membrane protein of the SHO1 pathway, namely Opy2, through their TM domains (see Fig. 9a for the structure of Opy2). To first determine if the TM domain of Opy2 is functionally important for osmostress-induced activation of Hog1 or not, we screened for hyperactive Opy2 mutants (for details, see the Methods section). Two hyperactive Opy2 mutations in the TM domain (F96I and A104V) strongly activated Hog1 when overexpressed (without osmostress) in *STE11-Q301P* host cells (Fig. 9b left panel). In *STE11-WT* host cells, however, Opy2-F96I only weakly activated Hog1, and Opy2-A104V did not activate Hog1 at all (Fig. 9b right panel). The Opy2-F96I A104V double mutant potently activated Hog1 even in *STE11-WT* cells, suggesting that F96I and A104V activated Hog1 by different mechanisms, and their effects are synergistic.

We then tested if Opy2 bound Sho1. Opy2–Sho1 binding was undetectable when Triton-X-100 or NP-40 were used for cell lysis, but became detectable if milder detergents (such as digitonin, Brij 97 or Brij L23) were used (Supplementary Fig. 6a). Opy2-A104V had a substantially higher affinity for Sho1 than did Opy2-WT or Opy2-F96I (Fig. 9c). To test if the increased affinity of Opy2-A104V for Sho1 was responsible for its hyperactivity, we isolated intragenic suppressor mutants of Opy2-A104V. The best mutant so far identified, I100D, completely suppressed the hyperactivity of Opy2-A104V (Fig. 9d). Importantly, the Opy2-I100D mutant was incapable of binding to Sho1 (Fig. 9e). Thus, enhanced Opy2–Sho1 interaction promotes Hog1 activation, likely by increasing the chance of direct Ste50–Sho1 interaction (Fig. 9f). Osmostress, however, did not increase Opy2–Sho1 interaction (Supplementary Fig. 6b).

We next examined which of the four Sho1 TM domains interacted with the Opy2 TM domain. The Sho1 TM2/TM3 trimer interface was not required for Opy2 binding, as the Sho1-WW mutant bound Opy2 as efficiently as Sho1-WT (Supplementary Fig. 6c). To more systematically identify the sites in Sho1 that interacted with Opy2, we employed the site-directed chemical crosslinking method. For that purpose, we generated single-Cys substitution mutants of Opy2ΔSR1-myc at residues near the extracellular end of the TM domain. Each single-Cys Opy2 mutant was expressed together with HA-Sho1* single-Cys mutants chosen from the four TM domains (also near their extracellular ends). These mutants were crosslinked *in vivo* with BMH, and cell lysates were prepared using a Triton-X-100 buffer so that non-covalently associated Opy2 would not co-precipitate with Sho1. HA-Sho1* was immunoprecipitated, and the covalently bound Opy2-myc/HA-Sho1* heterodimer was detected by immunoblotting using anti-myc antibody (Supplementary Fig. 7a). Strongly crosslinkable Sho1 Cys residues were found only in TM1, L3 and TM4 regions (Supplementary Fig. 7b). For example, Sho1-A124C in TM4 was preferentially crosslinked to Opy2-I93C, -G95C and -F96C (Fig. 9g). We thus conclude that Opy2 binds to the TM1/TM4 interface of Sho1 (Fig. 9h). This finding raises the possibility that the hyperactive Opy2-F96I activates Sho1 by inducing a structural change in Sho1. Such a mechanism explains the synergistic effect of the F96I and the high affinity A104V mutations. However, direct demonstration of Sho1 activation (structural change or Ste50 binding) by Opy2-F96I is currently difficult because only a small fraction of Sho1 is bound to Opy2.

### Hkr1 and Opy2 interact through their extracellular domains

**Figure 10:**
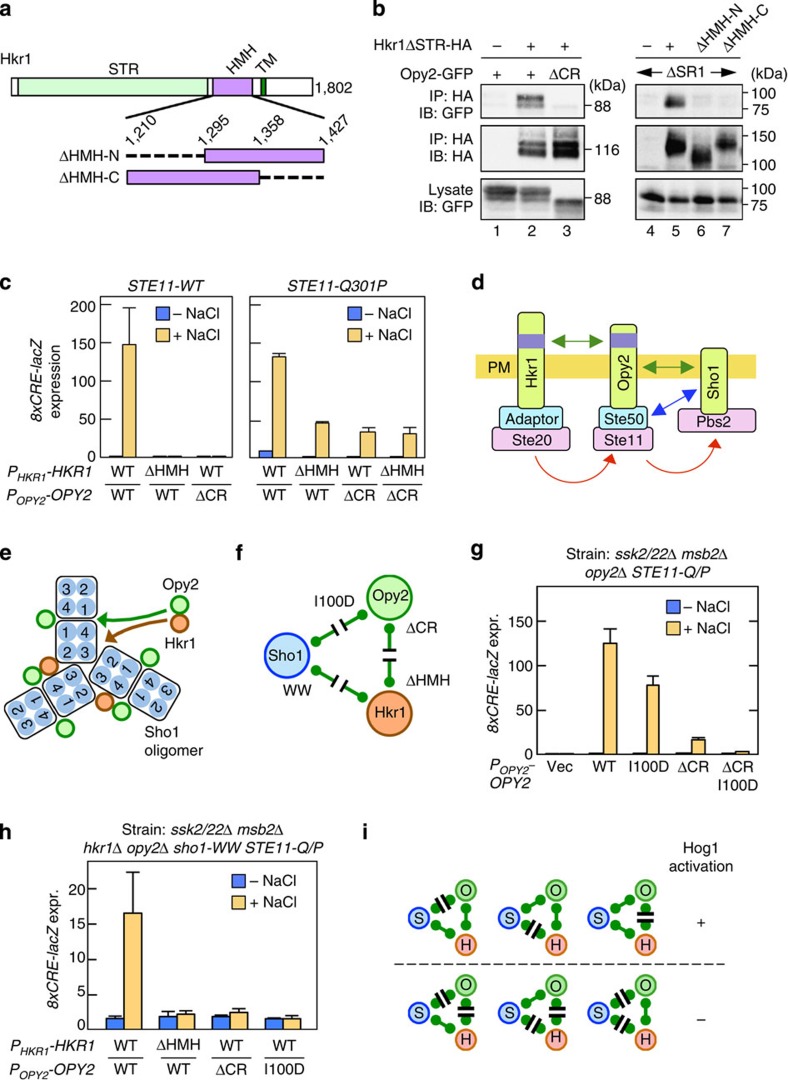
The roles of the Sho1–Opy2–Hkr1 interactions in osmotic activation of Hog1. (**a**) Schematic model of Hkr1. Structures of the HMH domain deletion mutants are enlarged. Numbers are amino-acid positions. (**b**) Opy2–Hkr1-binding assay. The indicated Hkr1 and Opy2 constructs were expressed in TM257 (left) or QG158 (right) strains. Cell lysates were prepared with Buffer A containing 1% digitonin. Hkr1ΔSTR-HA was immunoprecipitated, and co-precipitated Opy2-GFP was detected by immunoblotting. (**c**) Hog1-specific reporter assays of KY585 and KY599-2 strains co-transfected with the indicated Hkr1 and Opy2 expression plasmids. Cells were stimulated with or without 0.4 M NaCl for 30 min, and the expression of the *8xCRE-lacZ* reporter gene was determined. Error bars are s.d. (*n*⩾3). (**d**) Schematic model of sequential interaction among Hkr1, Opy2 and Sho1. (**e**) Summary of the interactions among Sho1, Opy2 and Hkr1. Only a portion of the Sho1 oligomer is shown. (**f**) Mutations that disrupt the interactions between Sho1, Opy2 and Hkr1. (**g**–**h**) Hog1-specific reporter assays of the yeast strains AN004 (**g**) and KY602-12 (**h**) transformed with expression plasmids for the indicated mutants of Opy2 and Hkr1 (native promoters). Cells were stimulated with or without 0.4 M NaCl for 30 min and expression of the *8xCRE-lacZ* reporter gene was determined. Error bars are s.d. (*n*=4). (**i**) Summary of the functional effects of mutations that disrupt interactions between Sho1 (S), Opy2 (O) and Hkr1 (H).

The binding of Sho1 to both Hkr1 and Opy2 suggested the possibility that Hkr1 and Opy2 might also interact. The HMH domain of Hkr1 is functionally essential for Hog1 activation^13^. We found that the Cys-rich (CR) domain of Opy2 is also essential for Hog1 activation (see below). As these domains are both extracellularly located (Fig. 10a, also see Fig. 9a), we examined the possibility that Hkr1 and Opy2 might interact through these domains. Indeed, Hkr1ΔSTR-HA and Opy2-GFP could be co-precipitated *in vivo* (Fig. 10b, lane 2), and this interaction was completely abolished by deletion of the CR domain from Opy2 (lane 3) or the HMH domain from Hkr1 (lanes 6–7). Thus, Hkr1 and Opy2 interact with each other in a manner that requires the Hkr1 HMH domain and the Opy2 CR domain.

In host cells in which only the HKR1 sub-branch is functional (*ssk2/22*Δ *msb2*Δ), neither Hkr1ΔHMH nor Opy2ΔCR could support Hog1 activation (Fig. 10c left panel), indicating that Hkr1–Opy2 binding is essential for osmostress-induced activation of the Hog1 MAPK cascade. However, both Hkr1ΔHMH and Opy2ΔCR could support substantial Hog1 activation if the cells expressed the constitutively active Ste11-Q301P (Fig. 10c right panel). These results imply that the Hkr1–Opy2 interaction is necessary for activation of Ste11 by Ste20. This conclusion is consistent with our previous finding that the Hkr1 cytoplasmic domain functionally interacts with Ste20, presumably through an unidentified adaptor protein, and is necessary for activation of Ste11 by Ste20 (ref. 10). It is likely that the Hkr1–Opy2 interaction tethers Ste20 and Ste11 together to allow Ste20 activate Ste11, which then activates Pbs2 (Fig. 10d).

### Role of a Sho1–Opy2–Hkr1 complex in Hog1 activation

Interactions among Sho1, Opy2 and Hkr1 potentially form a large complex as shown in Fig. 10e. Thus, the sequential pair-wise interaction model in Fig. 10d might be incomplete, as it does not incorporate the direct Hkr1–Sho1 interaction. We therefore examined the effects of mutations that disrupted one of the three pair-wise interactions (Fig. 10f) on osmostress-induced activation of Hog1 in Ste11-Q301P mutant host cells. We used the constitutively active Ste11-Q301P mutant, to monitor the effects of these mutations on the activation steps after Ste20 has activated Ste11. Disruption of the Sho1–Opy2 interaction (by Opy2-I100D) or the Opy2–Hkr1 interaction (by Opy2ΔCR) only partially interfered with the osmostress-induced Hog1 activation (Fig. 10g). Similarly, disruption of the Sho1–Hkr1 interaction alone (by sho1-WW encoded in the host genome) did not prevent Hog1 activation (Fig. 10h). In contrast, disruption of any two interactions simultaneously (for example, by Opy2ΔCR-I100D) completely abolished Hog1 activation. Thus, the disruption of any one interaction, among Sho1, Opy2 and Hkr1, had only moderate impact on Hog1 activation, whereas disruption of any two interactions completely inhibited Hog1 activation (Fig. 10i).

On the basis of these results, we conclude that the Sho1 oligomer serves, not only as an osmosensor, but also as a scaffold onto which the Opy2–Ste50–Ste11 and Hkr1–Ste20 subcomplexes bind, resulting in efficient signalling between these molecules. Disruption of any one of these interactions would be tolerated because the other two interactions would allow, if not very efficiently, the incorporation of all components into the signalling complex. Disruption of any two interactions would exclude at least one component from the complex, and prevent efficient Hog1 activation.

## Discussion

In this article, we demonstrated that Sho1 serves as an osmosensor as well as a scaffold. However, the two functions are not totally interdependent: the Sho1-WW mutant, which cannot oligomerize and cannot bind Hkr1, is nevertheless an efficient osmosensor. The recognition that Sho1 has two separable functions helps to unravel the puzzling complexity of the Sho1 mutant phenotypes. For example, *sho1* mutants are defective in the filamentous growth Kss1 MAPK signalling pathway, in which the osmosensing function of Sho1 is obviously irrelevant^31^. It is likely that only the scaffold function of Sho1 is involved in the filamentous growth pathway.

Although we presented evidence that Sho1 is an osmosensor, it does not necessarily imply that Sho1 is the only osmosensor in the SHO1 branch. As noted previously, the Myr–Sho1 mutant, which lacks the four Sho1 TM domains, could activate the Hog1 MAPK cascade in the presence of Msb2 (ref. 13). Because Myr–Sho1 cannot function as an osmosensor according to our model, we have proposed that Msb2 is also an osmosensor that functions in the SHO1 branch^13^. The highly glycosylated STR domain of Msb2, which inhibits spontaneous activation of Hog1, may serve as an osmosensing device. If so, the structurally similar Hkr1 might also function as an osmosensor. Here we propose a two-sensor mechanism in which the Hkr1 osmosensor activates the Ste20→Ste11 reaction (step 1), and the Sho1 osmosensor activates the Ste11→Pbs2 reaction (step 2; see Fig. 2a). Successful activation of Hog1 would occur only when both osmosensors were turned on: in other words, the HKR1 sub-branch would act as a logic AND gate. Because Hkr1 and Sho1 likely respond to different aspects of osmostress, the AND gate mechanism will ensure that a non-osmostress stimulus that might activate either Hkr1 or Sho1, but not both, will not activate Hog1. Substantiation of the two-sensor model requires further characterization of the Hkr1 putative osmosensor.

The 4TM protein Sho1 oligomerizes by the dimers-of-trimers architecture. Many other 4TM proteins, such as mammalian tetraspanins, are also known to oligomerize^32^,^33^,^34^,^35^,^36^. As other 4TM proteins might also oligomerize through repetitive TM–TM interactions similar to that of Sho1, Cys chemical crosslinking could be a valuable strategy for study of their structural organization.

Higher-order assemblies of TM signalling proteins are thought to be important for signal amplification, hypersensitivity, reduction of biological noise and spatial regulation^37^. Indeed, the SHO1 branch displays a steeply sigmoidal response to increasing osmolarity (that is, hypersensitivity)^12^, and has a low basal signalling (that is, noise reduction)^38^. It is plausible that the formation of a multi-component complex around the Sho1 oligomer is important to achieve these signalling characteristics of Sho1. Therefore, Sho1 will be an excellent model for investigation of the emerging paradigm of higher-order assembly in signal transduction.

## Methods

### Media and buffers

Standard yeast media and genetic procedures were previously described^39^,^40^. CAD medium consists of 0.67% yeast nitrogen base (Sigma), 2% glucose, 0.5% casamino acid (BD) and appropriate supplements (20 μg ml^−1^ uracil and 40 μg ml^−1^ tryptophan) as needed. CARaf and CAGal media are the same as CAD, except that they contain 2% raffinose or 2% galactose, respectively, in place of glucose. SRaf medium consists of 0.67% yeast nitrogen base and 2% raffinose with an appropriate yeast synthetic drop-out medium supplement. Buffer A for co-immunoprecipitation assays contains 50 mM Tris-HCl (pH 7.5), 15 mM EDTA, 15 mM EGTA, 2 mM dithiothreitol, 1 mM phenylmethylsulfonyl fluoride, 1 mM benzamidine, 5 μg ml^−1^ leupeptin, 50 mM NaF, 25 mM β-glycerophosphate and 150 mM NaCl. Buffer C2 for detergent-free membrane preparation contains 50 mM Tris-HCl (pH 7.2), 15 mM EDTA, 15 mM EGTA, 150 mM NaCl, 1 mM phenylmethylsulfonyl fluoride, 1 mM benzamidine and 5 μg ml^−1^ leupeptin. Buffer X for crosslinking contains 50 mM Tris-HCl (pH 7.2) and 15 mM EDTA. Buffer Z for β-galactosidase assay contains 60 mM Na_2_HPO_4_, 40 mM NaH_2_PO_4_, 10 mM KCl and 1 mM MgSO_4_, adjusted to pH 7.0. SDS loading buffer (1 × ) contains 50 mM Tris-HCl (pH 6.8), 2% SDS, 0.01% Bromophenol Blue, 10% glycerol and 700 mM 2-ME. For non-reducing conditions, 2-ME was omitted.

### Reagents

The following reagents were used. Cys-specific chemical crosslinkers: BMH and TMEA (Thermo Scientific), *o*-PDM and *p*-PDM (Sigma) and Bis-MTS (methanethiosulfonate) reagents (M1M through M17M) (Toronto Research Chemicals). Detergents: Brij L23, Brij 97 and Brij 99 (Sigma), Triton-X-100, NP-40 and Tween-20 (MP Biochemical) and Digitonin (Sigma and Calbiochem). *o*-phenanthroline (Wako). Polyene antibiotics: nystatin (Wako) and natamycin (Fluka). Other chemicals were purchased from Sigma, Wako Pure Chemical, Nacalai Tesque and BD.

### Yeast strains

All yeast mutants used in this work are derivatives of the S288C strain (Supplementary Table 1).

### Plasmid constructs

Deletion and missense mutants were constructed using PCR-based oligonucleotide mutagenesis, and were confirmed by nucleotide sequence determination.

*Vector plasmids*. pRS414, pRS416, YCpIF16, p414GAL1, p424GAL1, p416GAL1, p426GAL1 and p426TEG1 have been described^41^,^42^,^43^.

*Sho1 plasmids*. pRS416-Sho1 (=*P*_*SHO1*_*-SHO1, URA3, CEN6*) is a full-length *SHO1* genomic DNA clone, and expresses Sho1 under the control of the *SHO1* promoter. p416GAL1-Sho1-GFP (=*P*_*GAL1*_*-SHO1-GFP*, *URA3*, *CEN6*) expresses C-terminally GFP-tagged Sho1 (Sho1-GFP) under the control of the *GAL1* promoter. p424GAL1-GST-Sho1 (=*P*_*GAL1*_*-GST-SHO1, TRP1, 2*μ), p416GAL1-GST-Sho1 (=*P*_*GAL1*_*-GST-SHO1, URA3, CEN6*) and p426GAL1-GST-Sho1 (=*P*_*GAL1*_*-GST-SHO1, URA3, 2*μ) express N-terminally GST-tagged Sho1 (GST-Sho1) under the control of the *GAL1* promoter. YCpIF16-Sho1 (=*P*_*GAL1*_-*HA-SHO1, TRP1, CEN4*) encodes N-terminally HA-tagged Sho1 (HA-Sho1) under the control of the *GAL1* promoter.

Opy2 plasmids. pRS416-Opy2 (=*P*_*OPY2*_*-OPY2, URA3, CEN6*) is a full-length *OPY2* genomic DNA clone, and expresses Opy2 under the control of the *OPY2* promoter. p414GAL1-Opy2 (=*P*_*GAL1*_*-OPY2*, *TRP1*, *CEN6*) and p416GAL1-Opy2 (=*P*_*GAL1*_*-OPY2*, *URA3*, *CEN6*) express Opy2 under the control of the galactose-inducible *GAL1* promoter. p416GAL1-Opy2-GFP (=*P*_*GAL1*_*-OPY2-GFP*, *URA3*, *CEN6*) encodes C-terminally GFP-tagged Opy2 under the control of the *GAL1* promoter. p416GAL1-Opy2ΔSR1-myc (=*P*_*GAL1*_*-OPY2*Δ*SR1-3xmyc*, *URA3*, *CEN6*) encodes C-terminally 3 × myc-tagged Opy2ΔSR1 under the control of the *GAL1* promoter.

*Ste11 plasmid*. YCpIF16-Ste11ΔC (=*P*_*GAL1*_-*HA-STE11(1–413), TRP1, CEN4*) encodes N-terminally HA-tagged Ste11ΔC, which lacks the kinase catalytic domain, under the control of the *GAL1* promoter.

*Ste50 plasmids*. pRS414-Ste50 (=*P*_*STE50*_*-STE50, TRP1, CEN6*) is a full-length *STE50* genomic DNA clone, and expresses Ste50 under the control of the *STE50* promoter. p426TEG1-Ste50 (=*P*_*TEF2*_*-GST-STE50*, *URA3*, *2*μ) expresses N-terminally GST-tagged Ste50 (GST-Ste50) under the control of the *TEF2* promoter.

*Hkr1 plasmid*. pRS414GAL1-Hkr1ΔSTR-2xHA (=Hkr1Δ(41–1200)-HA, *TRP1*, *CEN6*) has two HA-tags, one at the site of internal deletion, and another at the C terminus.

*Other plasmids*. The cysteine-free GST mutant (GST*) was generated by mutating all four of the cysteines in GST (Cys85, Cys138, Cys169 and Cys178) to serine. GST* is functional^44^. Activation of the mating pheromone MAPK pathway was assayed using the mating pheromone-specific reporter pFUS1-lacZ (*FUS1-lacZ, URA3, CEN*) (=pSB231)^19^,^45^.

### HOG reporter assay

Yeast strains were transformed with either pRS414–8 × CRE-lacZ (=*8xCRE-lacZ, TRP1*, *CEN6*) or pRS416-8xCRE-lacZ (=*8xCRE-lacZ, URA3*, *CEN6*). Exponentially growing transformants were induced for reporter expression as described in each figure legend. To quantitatively assay β-galactosidase activity, 0.5 ml of culture was pelleted down, washed once and resuspended in 0.1 ml Z buffer. Cells were permeabilized by two cycles of freezing and thawing. Assay reaction was initiated by adding 0.7 ml of Z buffer containing 50 mM 2-ME and 0.16 ml of 4 mg ml^−1^ o-nitrophenyl β-D-galactopyranoside, and was incubated at 37 °C until mild yellow colour had developed. Following quenching of the reaction with 0.4 ml of 1 M Na_2_CO_3_, samples were centrifuged at 18,000*g* for 10 min at 4 °C, and the OD_420_ of the supernatants were measured. β-galactosidase activity was calculated using the following formula: β-galactosidase activity (Miller unit)=1,000OD_420_/(incubation time (_min_) × volume of the culture (ml) × OD_600_ of culture)^46^. All reporter assays were carried out in triplicate (or more) using independent cultures.

### *In vivo*-binding assay

Cells containing galactose-inducible expression constructs were exponentially grown in CARaf, and were cultured for an additional 2 h after addition of 2% galactose. Cells were harvested, washed once with ice-cold buffer A without detergent and were resuspended in 0.5 ml of buffer A. Cells were broken by vortexing with glass beads, and were centrifuged at 10,000 r.p.m. for 10 min at 4 °C in a microcentrifuge, and supernatant (cell extract) was recovered. An aliquot of cell extract (200–750 μg) was incubated with 50 μl of glutathione Sepharose beads for 2 h at 4 °C, washed three times in Buffer A and resuspended in SDS loading buffer. Samples were either incubated at 50 °C (when Sho1 was analysed) or boiled for 5 min, and separated by SDS–PAGE (polyacrylamide gel electrophoresis). Proteins were detected by immunoblotting. For immunoprecipitation and/or immunoblotting, the following antibodies were used as indicated: anti-HA 12CA5 (Roche, No. 11583816001) 200 ng ml^−1^; anti-HA 3F10 (Roche, No. 11867431001) 4 μg ml^−1^; anti-HA F-7 (Santa Cruz, sc-7392) 1:1,000 dilution; anti-GST B-14 (Santa Cruz, sc-138) 1:1,000 dilution; anti-GFP B-2 (Santa Cruz, sc-9996) 1:300 dilution; anti-myc 9E10 (Santa Cruz, sc-40) 1:1,000 dilution; anti-phospho-p38 (Cell Signaling Technology, No. 9211L) 1:1,000 dilution; and anti-Hog1 yC-20 (Santa Cruz, sc-6815) 1:1,000 dilution. Images were digitally captured using ChemiDoc XRS Plus (Bio-Rad) equipped with a charge-coupled device camera. Full scans of representative immunoblots are presented in Supplementary Figs 8 and 9.

### Isolation of constitutively active OPY2 mutants

A DNA segment including the *OPY2* coding region was mutagenized by error-prone PCR in the presence of 0.1 or 0.2 mM MnCl_2_. KT018 (*ssk2*Δ *ssk22*Δ *STE11-Q301P*) cells carrying an attenuated *8xCRE-lacZ* reporter gene (pRS416-8xCRE-CYC^m^-lacZ) were co-transformed with the PCR products and linearized pRS414-PGAL1. Gap-repaired plasmids were selected on CAD (w/o Ura and Trp) plates, replica-plated onto nitrocellulose membrane disks, and incubated on CAGal plates overnight to induce the plasmid-borne *OPY2* gene. Activation of the HOG MAPK pathway was assessed qualitatively using a colony-lift β-galactosidase filter assay^39^. Constitutively active *OPY2* mutants that induced *lacZ* expression, which rendered the cells blue, were recovered.

### Preparation of membrane fractions

The appropriate strain, for example, KT079, carrying an HA-Sho1* expression plasmid YCpIF16-Sho1*, was grown to log phase in a selective medium. Expression of the HA-Sho1* mutant protein was induced for 2 h with 2% galactose. The membrane fraction was then prepared as follows. In brief, 20 ml of culture was centrifuged at 2,000*g* for 3 min, and the cell pellet was resuspended in 500 μl of the detergent-free Buffer C2. Cells were broken by vigorous vortexing with glass beads. Unbroken cells were removed by two rounds of low-speed centrifugation (300*g* for 3 min), and the supernatant fraction (S300) was subjected to a high-speed centrifugation (13,000*g* for 10 min). The precipitated fraction (P13,000) was resuspended in 150 μl of Buffer X.

### Chemical crosslinking of membrane fractions

Chemical crosslinkers were dissolved in dimethyl sulfoxide (DMSO) or *N*,*N*-dimethylformamide (DMF). In a typical chemical crosslinking reaction, 30 μl of the membrane fraction was incubated with 0.2 mM crosslinker (or control DMSO or DMF) for 20 min at 25 °C. Immediately after the reaction, 15 μl 3 × SDS loading buffer (containing 2-ME) was added and incubated for 5 min at 37 °C. Samples were subjected to SDS–PAGE under reducing conditions (non-reducing conditions in the case of MTS-crosslinkers such as M1M), and HA-Sho1* was detected by immunoblotting.

### Chemical crosslinking of Sho1 in intact cells

A chemical crosslinker (or the control DMSO or DMF) was added directly to an exponentially growing yeast culture. Alternatively, the yeast culture was mixed with an equal volume of fresh medium that contained an appropriate concentration of crosslinkers. After incubation at 30 °C for 5 min (or as specified in the legends), cells were centrifuged at 1,700*g* for 3 min. To quench the crosslinking reaction, cell pellets were resuspended in 1 ml Buffer C2 containing 50 mM dithiothreitol, and incubated on ice for 15 min. Cells were then precipitated by a brief spin in a microcentrifuge tube, frozen in liquid N_2_ and thawed and resuspended in 400 μl Buffer C2. The membrane fraction was prepared and subjected to immunoblot analyses.

## Author contributions

K.T., K.Y. and H.S. designed the project. K.T., K.Y., M.N., T.T., A.N., M.S. and T.M. performed the experiments. All authors contributed to data analysis and writing of the paper.

## Additional information

**How to cite this article:** Tatebayashi, K. *et al*. Osmosensing and scaffolding functions of the oligomeric four-transmembrane domain osmosensor Sho1. *Nat. Commun.* 6:6975 doi: 10.1038/ncomms7975 (2015).

## Supplementary Material

Supplementary Information Supplementary Figures 1-9, Supplementary Table 1 and Supplementary References.

## Figures and Tables

**Figure 1:**
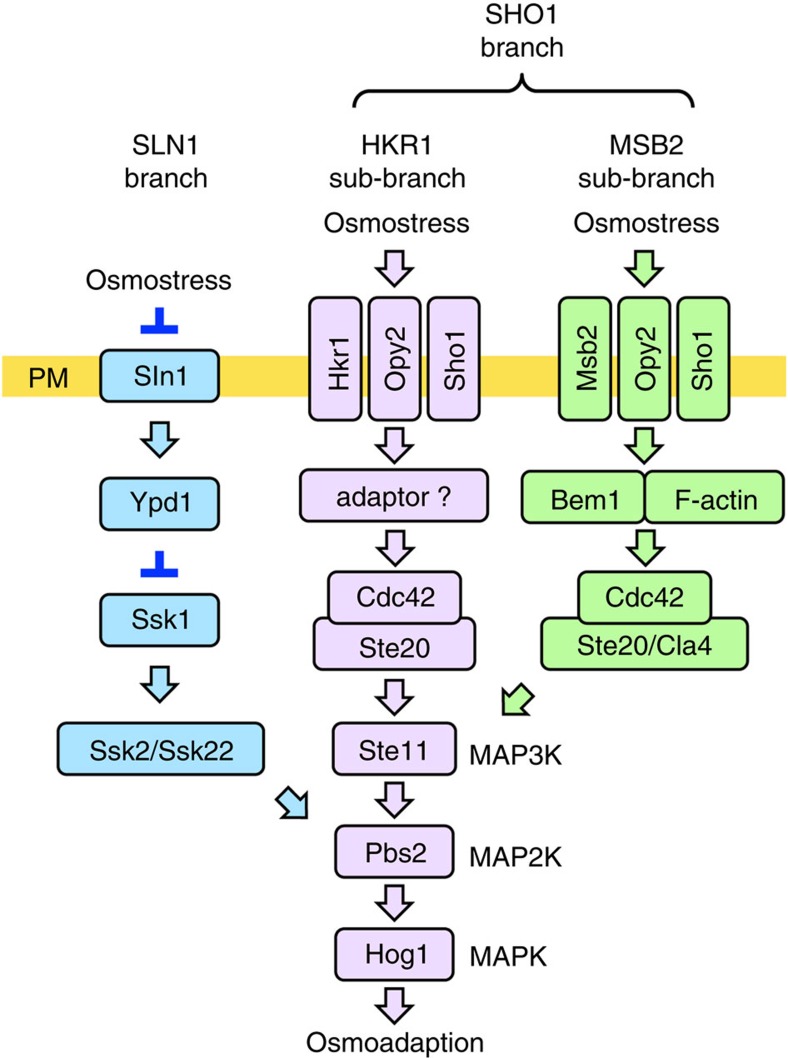
A schematic model of the HOG pathway. Proteins that are involved in the HRK1 sub-branch are shown in lavender. Proteins that are specific to the SLN1 branch are coloured blue and those that are involved in the MSB2 sub-branch are coloured green. The proteins separated by a slash (/) are functionally redundant. Not all the known components are shown. The yellow horizontal bar represents the plasma membrane (PM). Arrows indicate activation, whereas the inverted T-shaped bars represent inhibition.
