# Supplementary material for: Osmosensing and scaffolding functions of the oligomeric four-transmembrane domain osmosensor Sho1
Source: Nat Commun. 2015 Apr 21;6:6975. doi: 10.1038/ncomms7975 (PMC4411306; doi:10.1038/ncomms7975)
Supplement: Supplementary Information — Supplementary Figures 1-9, Supplementary Table 1 and Supplementary References. [file ncomms7975-s1.pdf]

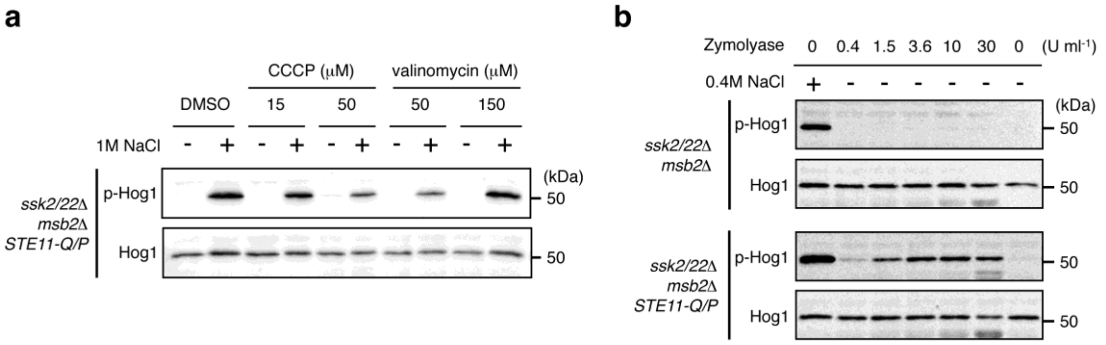

Supplementary Figure 1. Enzymatic digestion of the cell wall activates the Sho1 osmosensor.

(**a**) Leakage of K^+ or H^+ does not activate the Sho1 osmosensor. The yeast strain KT033 (*ssk2Δ ssk22Δ mbs2Δ STE11-Q301P*) was treated with the indicated concentrations of the proton ionophore carbonylcyanide m-chlorophenylhydrazone (CCCP), the potassium ionophore valinomycin, or the vehicle DMSO alone, for 5 min at 30 °C. Cells were then stimulated with (+) or without (-) 1 M NaCl for a further 5 min. Hog1 phosphorylation and total Hog1 were determined by immunoblotting using, respectively, anti-phospho-p38 antibody and anti-Hog1 yC20 antibody.

(**b**) Enzymatic digestion of the cell wall activates the Sho1 osmosensor. The yeast strains KT034 (*ssk2Δ ssk22Δ mbs2Δ*) and KT033 were incubated with the indicated concentrations of Zymolyase-100T for 15 min, or with 0.4 M NaCl (as a positive control) for 10 min, at 25 °C. Hog1 phosphorylation and total Hog1 were determined as in (a).

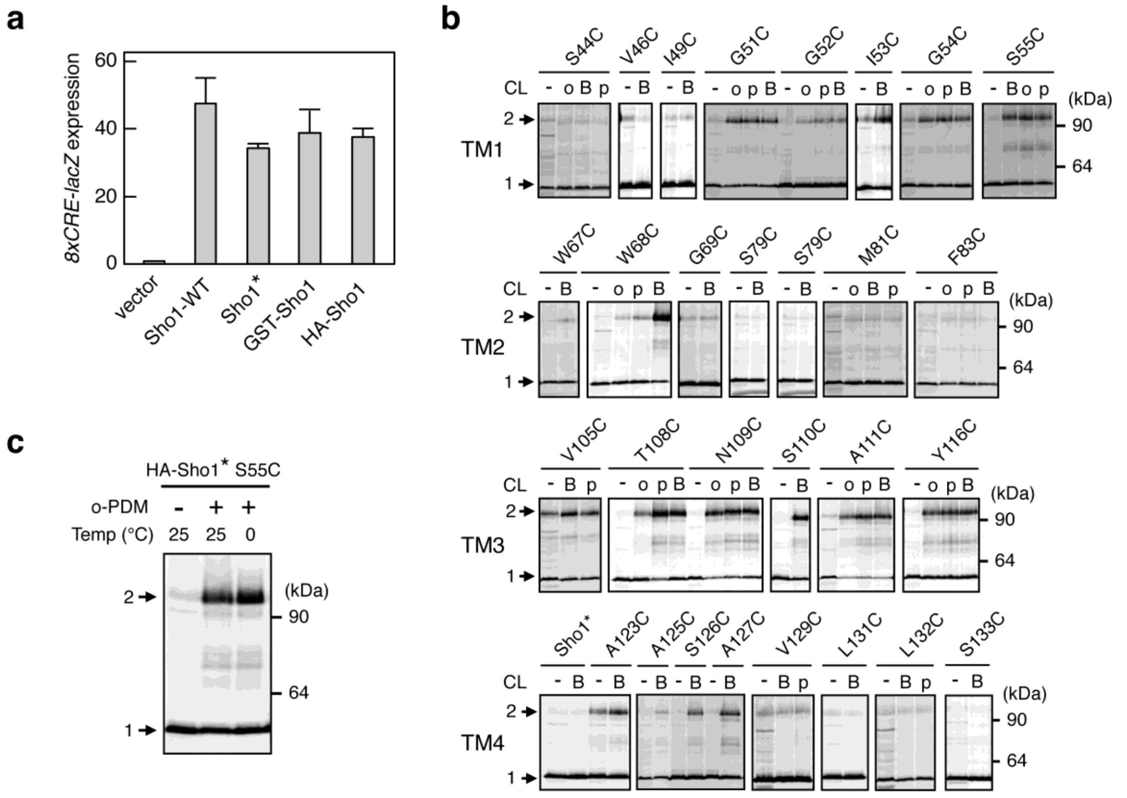

Supplementary Figure 2. Chemical crosslinking analyses of Sho1 oligomers.

(a) Abilities of the Sho1 constructs used in this study to activate Hog1 upon osmostress. The yeast strain KT079 (*ssk2/22Δ sho1Δ*) was co-transformed with the Hog1 reporter plasmid pRS414-8xCRE-lacZ together with the indicated derivatives of pRS416-Sho1. Exponentially growing cells were exposed to osmostress (0.4 M NaCl) for 30 min and expression of the Hog1-specific reporter gene *8xCRE-lacZ* was assayed. Error bars represent SD (n=3).

(b) Chemical crosslinking of Sho1 single-Cys substitution mutants. The indicated mutant derivatives of the Cys-less HA-Sho1* were individually expressed in KT079 cells. Isolated membrane fractions were treated with the indicated crosslinkers. Samples were subjected to SDS-PAGE (with 2-ME), and HA-Sho1* was detected by immunoblotting. Numbers on the right indicate the extent of Sho1 polymerization (1, monomer; 2, dimer). CL, crosslinker; o, *o*-PDM; p, *p*-PDM; B, BMH.

(c) Effect of temperature on Sho1 homo-crosslinking. Sho1*-S55C was crosslinked with 0.2 mM *o*-PDM for 20 min at either 25 °C or 0 °C and was then analyzed as in (b).

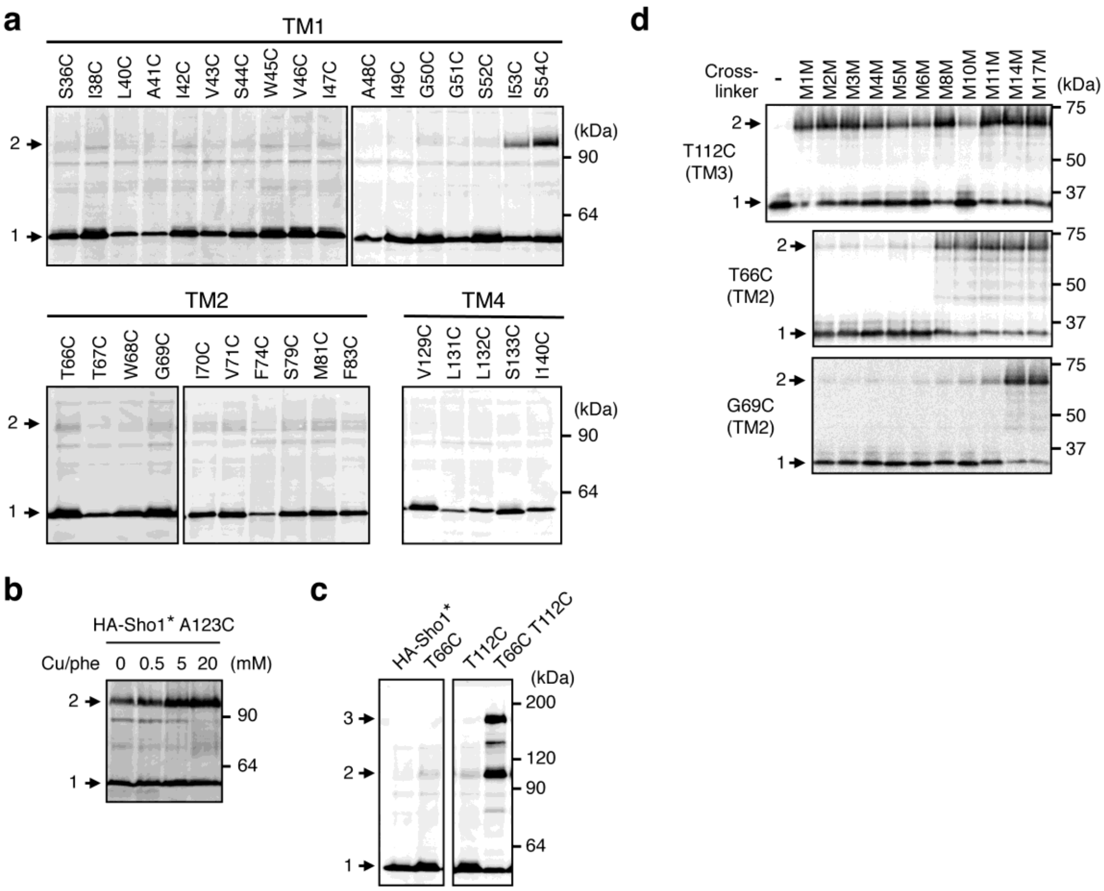

Supplementary Figure 3. Analyses of the structure of Sho1 oligomers.

(a) Spontaneous disulfide bond formation by Sho1 single-Cys substitution mutants. KT079 cells (*ssk2/22*Δ *sho1*Δ) were transformed with expression plasmids for the indicated mutant derivatives of HA-Sho1* under the control of the *GAL1* promoter. Isolated membrane fractions were subjected to SDS-PAGE (without 2-ME), and HA-Sho1* was detected by immunoblotting.

(b) Enhancement of disulfide bond formation by the oxidant copper/*o*-phenanthroline (Cu/phe). HA-Sho1* A123C was expressed in KT079 cells as in (a). Cells were treated with the indicated concentration of Cu/phe for 10 min at 30 °C, and were then subjected to immunoblotting analysis as in (a).

(c) Disulfide bond formation of a double-Cys Sho1 mutant. HA-Sho1* T66C and/or T112C was expressed in KT079, and spontaneous disulfide bond formation was analyzed as in (a).

(d) Chemical crosslinking by molecular rulers. The indicated single-Cys substitution mutants of HA-Sho1*Δ11 were individually expressed in KT079 cells. Isolated membrane fractions were treated with a set crosslinkers with different spacer lengths (M1M ~ M17M) *in vitro*. Samples were subjected to SDS-PAGE under non-reducing conditions. HA-Sho1* was detected by immunoblotting.

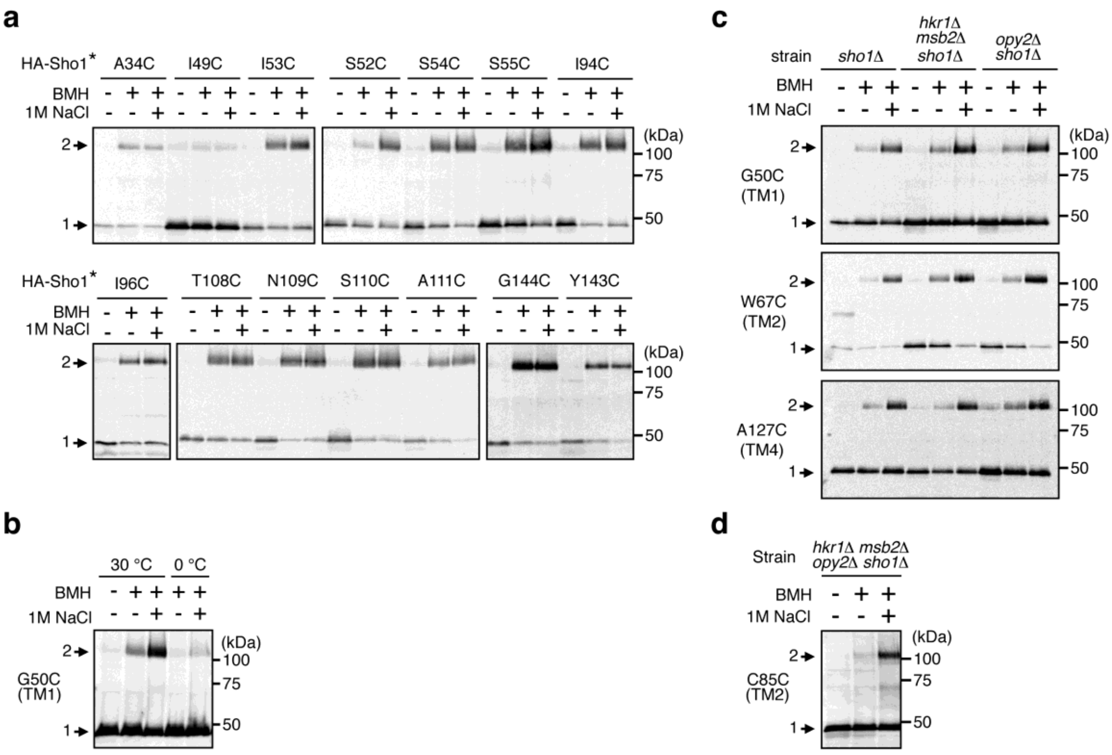

Supplementary Figure 4. Osmostress-induced structural changes in Sho1 oligomers.

(a) Effect of osmostress on chemical crosslinking of Sho1 single-Cys mutants. The yeast strain KT079 (*ssk2/22*Δ *sho1*Δ) was transformed with expression plasmids for the indicated derivatives of HA-Sho1* under the *GAL1* promoter. Expression of HA-Sho1* was induced by 2% galactose for 2 h, and HA-Sho1 was crosslinked in intact cells with 0.4 mM BMH for 10 min, in the presence (+) or absence (-) of 1 M NaCl.

(b) Effects of temperature on Sho1 crosslinking. HA-Sho1* G50C was expressed in KT079, and crosslinked as in (A) except at 30 °C and 0 °C**.**

(c-d) Effects of host genotypes on Sho1 crosslinking. The indicated derivatives of HA-Sho1* were expressed in KT079 (*ssk2/22*Δ *sho1*Δ), KT064 (*ssk2/22*Δ *hkr1*Δ *msb2*Δ *sho1*Δ), or KT094 (*ssk2/22*Δ *opy2*Δ *sho1*Δ) (c) or in KY594-1 (*ssk2/22*Δ *hkr1*Δ *msb2*Δ *opy2*Δ *sho1*Δ) (d). Crosslinking in the presence or absence of osmostress was conducted as in (a).

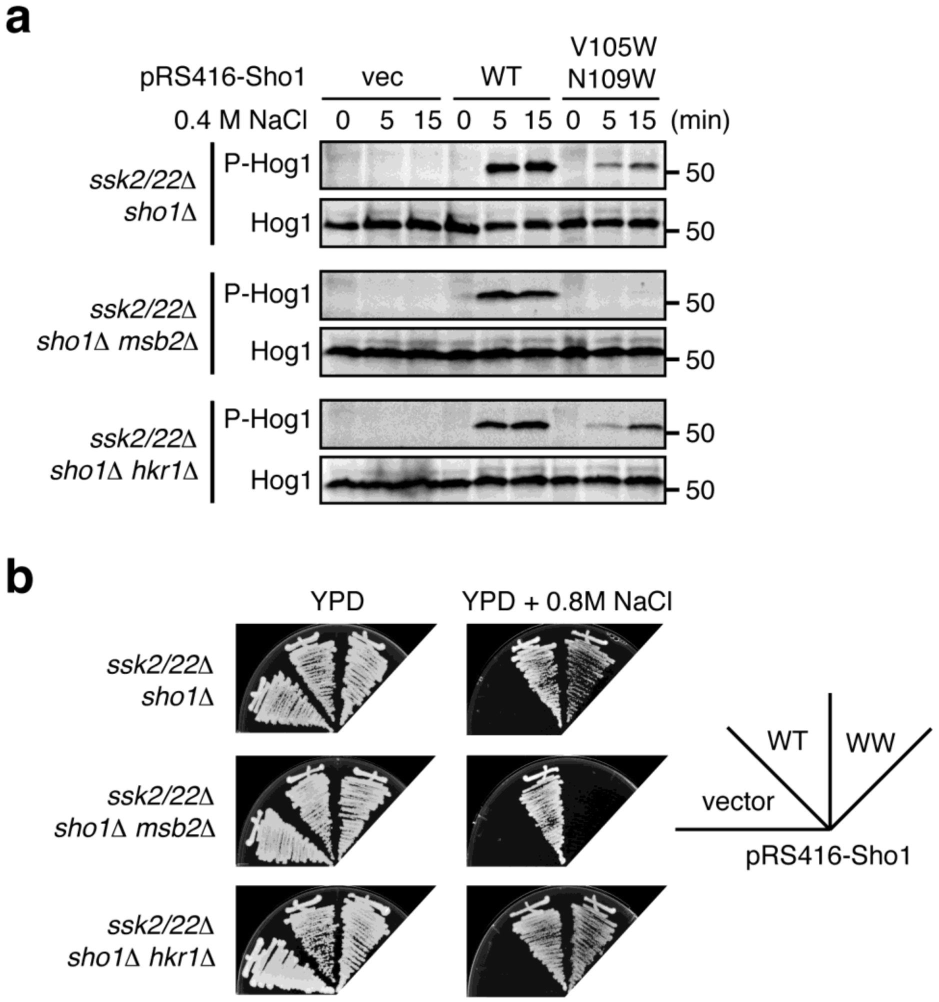

Supplementary Figure 5. Functional roles of the Sho1 TM2/TM3 interface.
(a-b) Phenotypes of the *SHO1* V105W N109W mutation. The yeast strains KT079 (*ssk2/22*Δ *sho1*Δ), KT053 (*ssk2/22*Δ *sho1*Δ *msb2*Δ), and KT088 (*ssk2/22*Δ *sho1*Δ *hkr1*Δ) were transformed with either the wild-type (WT) or the V105W N109W (WW) mutant of pRS416-Sho1, or the empty pRS416 vector (vec). (a) Effect of the WW mutation on Hog1 phosphorylation. Cells were treated with 0.4 M NaCl for the indicated times before preparation of the cell lysates. Phosphorylated Hog1 (P-Hog1) and total Hog1 were probed by immunoblotting. (b) Effect of the WW mutation on cell growth under high osmolarity stress. Cells were grown on the indicated agar plates.

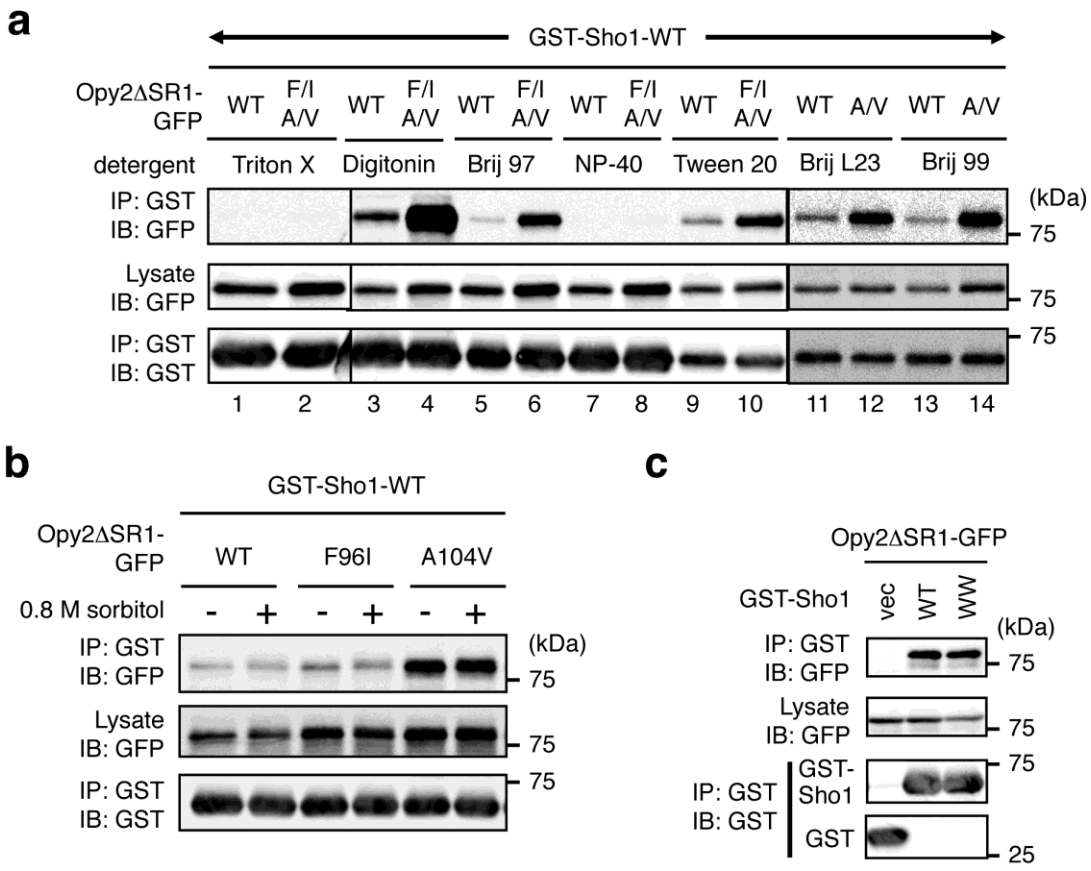

Supplementary Figure 6. Analyses of the Opy2-Sho1 interaction.

(a) Effects of detergents on Opy2-Sho1 co-precipitation. An expression plasmid that encoded GST-Sho1-WT, and another plasmid that encoded the indicated mutant of Opy2ΔSR1-GFP, both under the control of the *GAL1* promoter, were co-transformed into the host cell FP75 (*ssk2*Δ *ssk22*Δ *ste11*Δ). The ΔSR1 mutant of Opy2 was used because it migrates as an unglycosylated single band in SDS-PAGE [1], and a *ste11*Δ mutant strain was used to prevent any possible feedback response. After induction of protein expression with 2% galactose for 2 h (lanes 1-10) or 1 h (lanes 11-14), cell extracts were prepared using buffers that contained one of the following detergents: 0.1% Triton X-100, 1% Digitonin, 0.1% Brij 97, 0.1% NP-40, 0.1% Tween-20, 0.1% Brij L23 or 0.2% Brij 99. GST-Sho1 was affinity purified using glutathione Sepharose beads, and co-precipitated Opy2ΔSR1-GFP was detected by immunoblotting. F/I, F96I; A/V, A104V; WT, no mutation in the TM region.

(b) Effect of external high osmolarity on Opy2-Sho1 binding. Expression plasmids for GST-Sho1-WT (or the empty vector that express GST only) and the indicated mutants of Opy2ΔSR1-GFP were co-transformed into the host cell FP75 (*ssk2*Δ *ssk22*Δ *ste11*Δ). After induction of protein expression with 2% galactose for 2 h, cells were exposed to 0.8 M sorbitol for ~5 min, and cell extracts were prepared using a buffer containing Brij 97. GST-Sho1 was affinity purified, and co-precipitated Opy2ΔSR1-GFP was detected by immunoblotting.

(c) Opy2-Sho1 binding assays were conducted as in (A) except that Buffer A contained 1% digitonin. WW, V105W N109W.

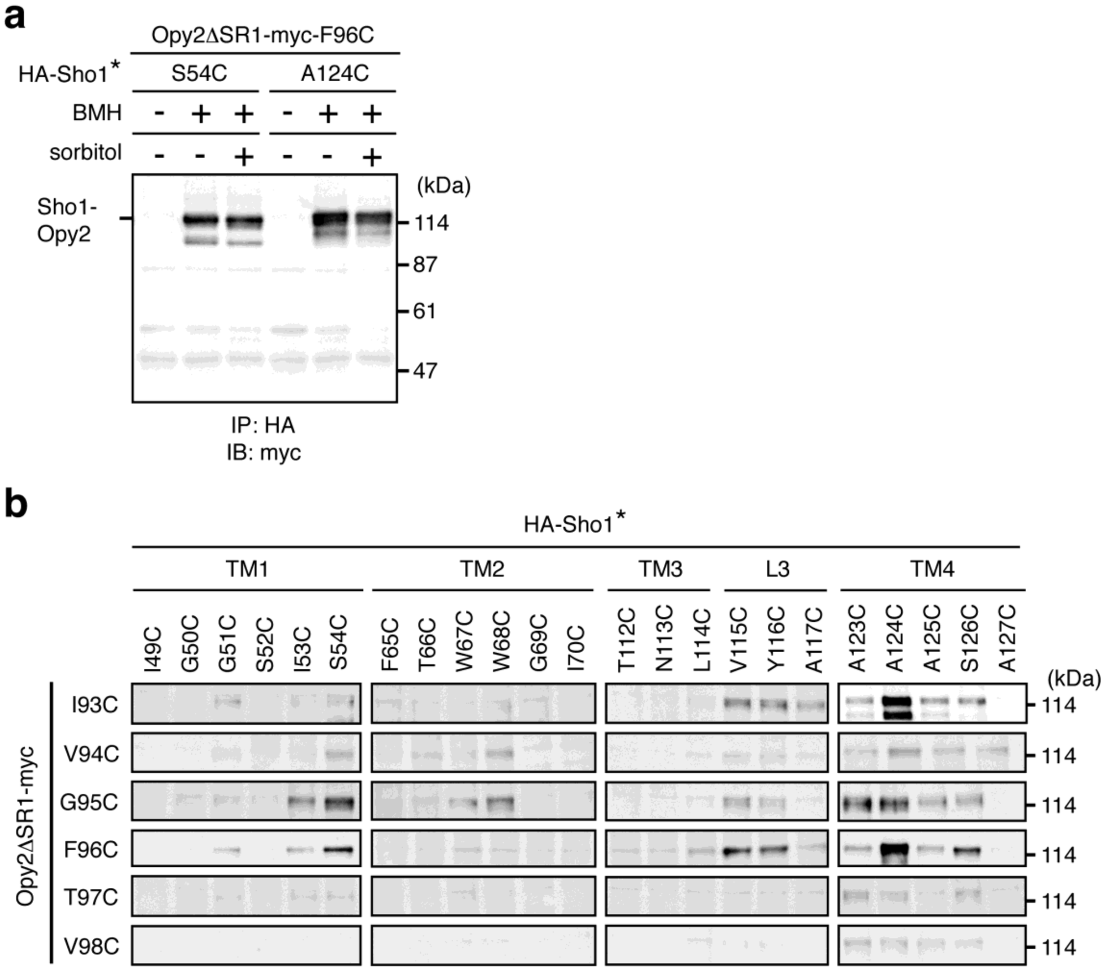

Supplementary Figure 7. Chemical crosslinking between Opy2 and Sho1.

(a) KY590-1 (*ssk2*Δ *ssk22*Δ *sho1*Δ *opy2*Δ) cells were co-transformed with the F96C mutant of p416GAL1-Opy2ΔSR1-myc (P_{GAL1}-*OPY2ΔSR1-3xmyc*) and either the S54C or A124C derivative of YCpIF16-Sho1* (P_{GAL1}-*HA-SHO1**). Cells were treated, simultaneously, with (+) or without (-) 0.8 M sorbitol and 0.4 mM BMH at 30 °C for 5 min. HA-Sho1* was immunoprecipitated (IP) and then immunoblotted (IB) with anti-myc antibody to detect the crosslinked Opy2-Sho1 heterodimers.

(b) KY590-1 cells co-transformed with one of the indicated derivatives of p416GAL1-Opy2ΔSR1-myc (shown on the left) and of YCpIF16-Sho1* (shown on the top) were analyzed as in (a). Only the portions of the gels that contain the crosslinked Opy2-Sho1 heterodimers are shown.

Fig. 2f

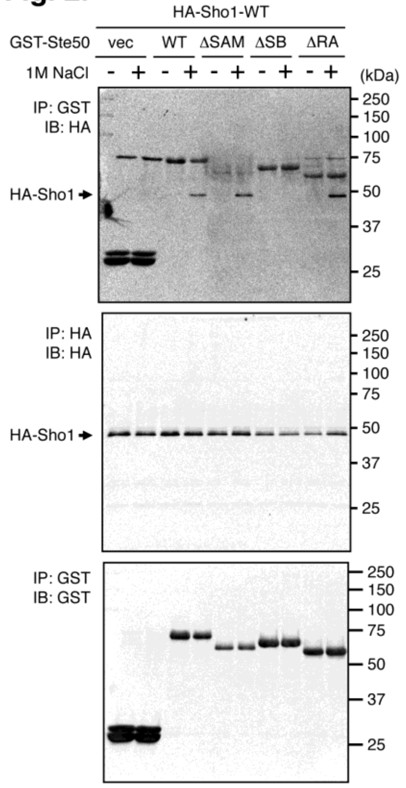

Fig. 4a

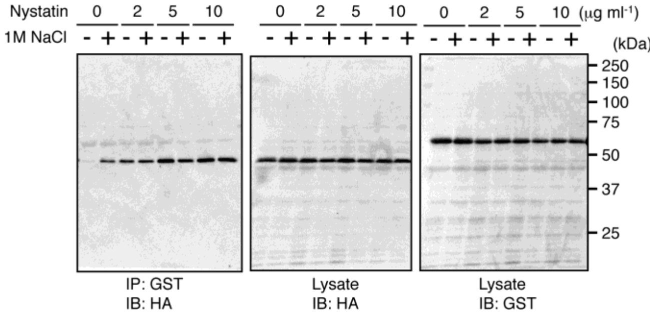

Fig. 4d

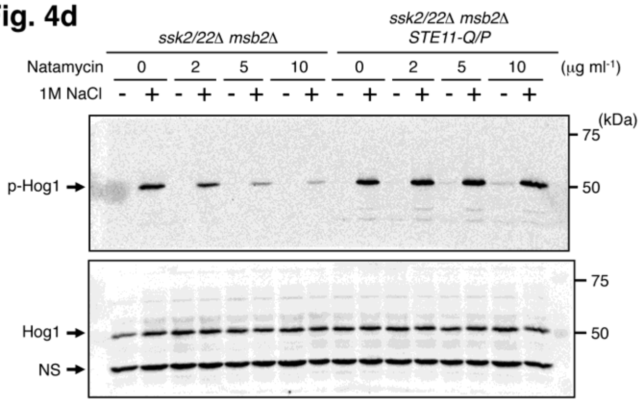

Fig. 5c

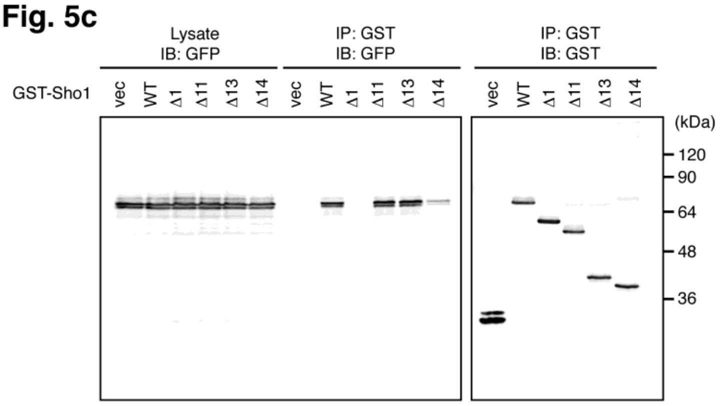

Fig. 6a (I94C)

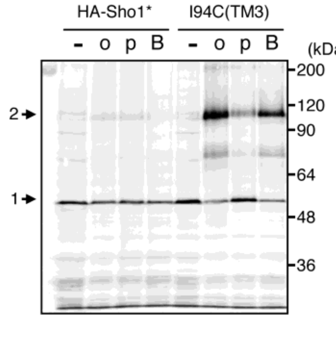

Supplementary Figure 8.

Uncropped scans of representative immunoblots in Figures 2, 4, 5 and 6.

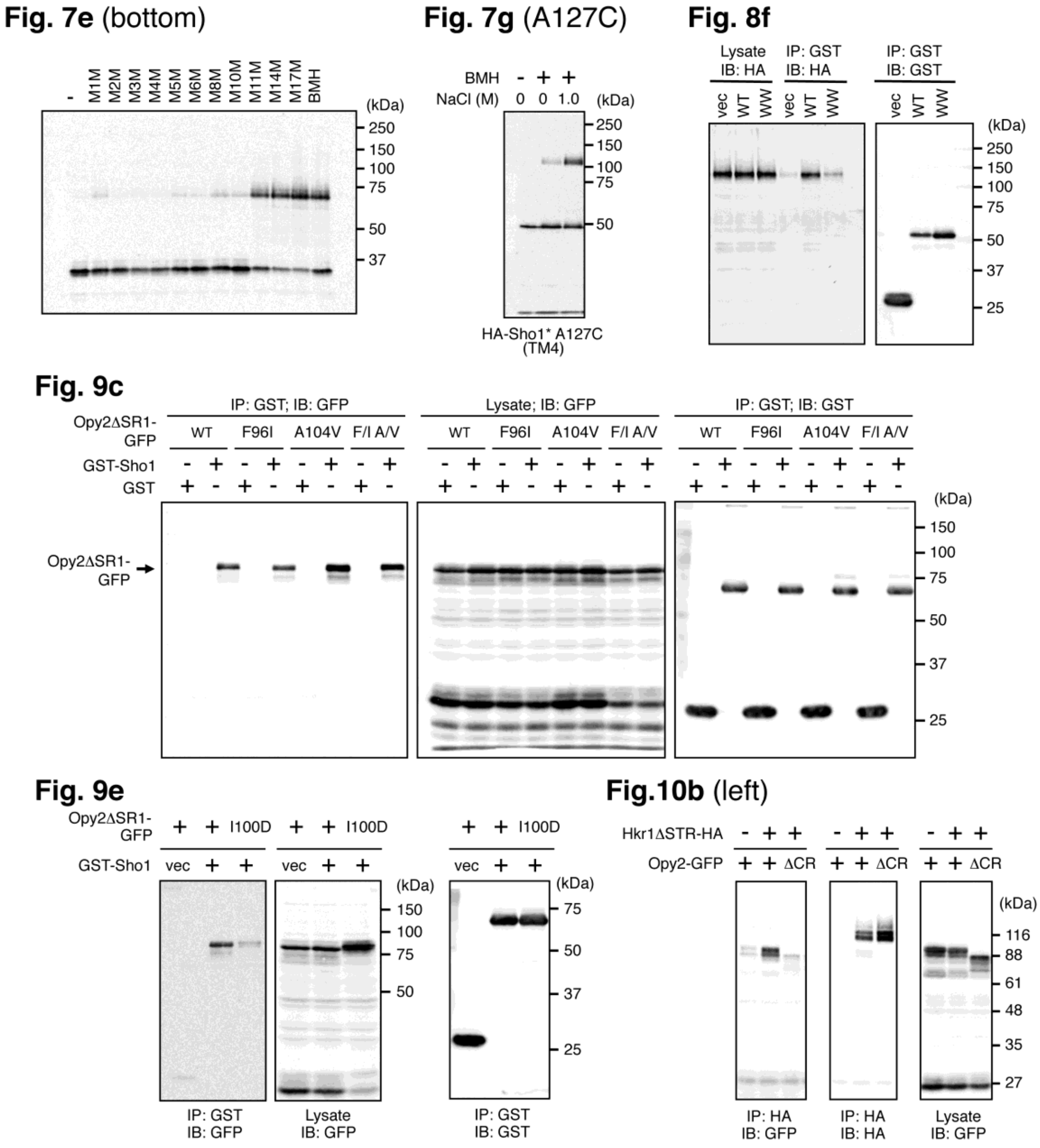

Supplementary Figure 9.

Uncropped scans of representative immunoblots in Figures 7-10.

Supplementary Table 1. Yeast strains used in this study.

Strain	Genotype	Source
AN001	*MATα ura3 leu2 trp1 his3 ssk2::LEU2 ssk22::LEU2 hkr1::natMX4 msb2::kanMX6 STE11-Q301P*	This study
AN004	*MATα ura3 leu2 trp1 his3 ssk2::LEU2 ssk22::LEU2 msb2::kanMX6 opy2::natMX4 STE11-Q301P*	This study
FP66	*MAT***a** *ura3 leu2 trp1 his3 ste50::HIS3*	F. Posas [2]
FP75	*MATα ura3 leu2 trp1 his3 ssk2::LEU2 ssk22::LEU2 ste11::HIS3*	F. Posas [2]
KT033	*MATα ura3 leu2 trp1 his3 ssk2::LEU2 ssk22::LEU2 msb2::kanMX6 STE11-Q301P*	This study
KT034	*MATα ura3 leu2 trp1 his3 ssk2::LEU2 ssk22::LEU2 msb2::kanMX6*	K. Tatebayashi [3]
KT040	*MATα ura3 leu2 trp1 his3 ssk2::LEU2 ssk22::LEU2 msb2::kanMX6 sho1::HIS3*	This study
KT048	*MAT***a** *ura3 leu2 trp1 his3 ssk2::hisG ssk22::hisG ste50::hisG*	This study
KT053	*MATα ura3 leu2 trp1 his3 ssk2::LEU2 ssk22::LEU2 msb2::kanMX6 sho1::hphMX4*	K. Tatebayashi [3]
KT064	*MATα ura3 leu2 trp1 his3 ssk2::LEU2 ssk22::LEU2 hkr1::natMX4 msb2::kanMX6 sho1::hphMX4*	K. Tatebayashi [3]
KT075	*MATα ura3 leu2 trp1 his3 ssk2::LEU2 ssk22::LEU2 hkr1::natMX4 msb2::kanMX6 pbs2::HIS3 sho1::hphMX4*	K. Tatebayashi [3]
KT079	*MATα ura3 leu2 trp1 his3 ssk2::LEU2 ssk22::LEU2 sho1::natMX4*	K. Tatebayashi [3]
KT088	*MATα ura3 leu2 trp1 his3 ssk2::LEU2 ssk22::LEU2 hkr1::natMX4 sho1::hphMX4*	K. Tatebayashi [3]
KT094	*MATα ura3 leu2 trp1 his3 ssk2::LEU2 ssk22::LEU2 opy2::kanMX6 sho1::hphMX4*	This study
KT193	*MAT***a** *ura3 leu2 trp1 his3 ssk2::hisG ssk22::hisG ste50::hisG msb2::natMX4*	This study
KT200-3	*MATα ura3 leu2 trp1 his3 ssk2::LEU2 ssk22::LEU2 hkr1::natMX4 msb2::kanMX6 sho1::hphMX4 STE11-Q301P*	This study

KY477	*MATα ura3 leu2 trp1 his3 ssk2::LEU2 ssk22::LEU2 opy2::kanMX6*	K. Tatebayashi [3]
KY517	*MATα ura3 leu2 trp1 his3 ssk2::LEU2 ssk22::LEU2 opy2::kanMX6 STE11-Q301P*	K. Yamamoto [4]
KY525	*MAT***a** *ura3 leu2 trp1 his3 ssk2::hisG ssk22::hisG fus3::kanMX6 kss1::hphMX4 hog1::LEU2*	K. Yamamoto [4]
KY585	*MATα ura3 leu2 trp1 his3 ssk2::LEU2 ssk22::LEU2 hkr1::hphMX4 msb2::kanMX6 opy2::natMX4*	This study
KY590-1	*MAT***a** *ura3 leu2 trp1 his3 ssk2::hisG ssk22::hisG opy2::natMX4 sho1::hphMX4*	This study
KY594-1	*MATα ura3 leu2 trp1 his3 ssk2::LEU2 ssk22::LEU2 hkr1::hphMX4 msb2::kanMX6 opy2::natMX4 sho1::hisG*	This study
KY598-3	*MATα ura3 leu2 trp1 his3 ssk2::LEU2 ssk22::LEU2 hkr1::natMX4 msb2::kanMX6 opy2::hphMX4 SHO1-P120L*	This study
KY599-2	*MATα ura3 leu2 trp1 his3 ssk2::LEU2 ssk22::LEU2 hkr1:hphMX4 msb2::kanMX6 opy2::natMX4 STE11-Q301P*	This study
KY602-12	*MATα ura3 leu2 trp1 his3 ssk2::LEU2 ssk22::LEU2 hkr1:hphMX4 msb2::kanMX6 opy2::natMX4 sho1-V105W/N109W STE11-Q301P*	This study
QG153	*MATα ura3 leu2 trp1 his3 ssk2::LEU2 ssk22::LEU2 sho1::HIS3*	Qingyuan Ge
QG158	*MAT***a** *ura3 leu2 trp1 his3 ssk2::hisG ssk22::hisG*	Qingyuan Ge
TM257	*MATα ura3 leu2 trp1 his3 ssk2::LEU2 ssk22::LEU2*	T. Maeda [5]

All strains were constructed in our laboratory, and are derived from S288C.

Supplemental references

1. Yang H-Y, Tatebayashi K, Yamamoto K, Saito H. Glycosylation defects activate filamentous growth Kss1 MAPK and inhibit osmoregulatory Hog1 MAPK. *EMBO J* **28**, 1380-1391 (2009).

2. Posas F, Witten EA, Saito H. Requirement of STE50 for osmostress-induced activation of the STE11 mitogen-activated protein kinase kinase kinase in the high-osmolarity glycerol response pathway. *Mol Cell Biol* **18**, 5788-5796 (1998).

3. Tatebayashi K*, et al.* Transmembrane mucins Hkr1 and Msb2 are putative osmosensors in the SHO1 branch of yeast HOG pathway. *EMBO J* **26**, 3521-3533 (2007).

4. Yamamoto K, Tatebayashi K, Tanaka K, Saito H. Dynamic cotrol of yeast MAP kinase network by induced association and dissociation between the Ste50 scaffold and the Opy2 membrane anchor. *Mol Cell* **40**, 87-98 (2010).

5. Maeda T, Takekawa M, Saito H. Activation of yeast PBS2 MAPKK by MAPKKKs or by binding of an SH3-containing osmosensor. *Science* **269**, 554-558 (1995).
